# CircMET promotes tumor proliferation by enhancing CDKN2A mRNA decay and upregulating SMAD3

**DOI:** 10.1186/s12943-022-01497-w

**Published:** 2022-01-18

**Authors:** Lei Yang, Yi Chen, Ning Liu, Yanwen Lu, Wenliang Ma, Zhenhao Yang, Weidong Gan, Dongmei Li

**Affiliations:** 1 Immunology and Reproduction Biology Laboratory & State Key Laboratory of Analytical Chemistry for Life Science, Medical School, Nanjing University, Nanjing, 210093 Jiangsu China; 2 Jiangsu Key Laboratory of Molecular Medicine, Nanjing University, Nanjing, 210093 Jiangsu China; 3 Department of Urology, Nanjing Drum Tower Hospital Clinical College of Traditional Chinese and Western Medicine, Nanjing University of Chinese Medicine, Nanjing, 210008 Jiangsu China; 4 Department of Urology, Nanjing Drum Tower Hospital, The Affiliated Hospital of Nanjing University Medical School, Nanjing, 210008 Jiangsu China

**Keywords:** circMET, m^6^A modification, NONO-TFE3, CDKN2A, SMAD3

## Abstract

**Background:**

Functions of CircMET (hsa_circ_0082002) which is a circular RNA and derived from *MET* gene remain understood incompletely. In the present study, Xp11.2 translocation/*NONO-TFE3* fusion renal cell carcinoma (*NONO-TFE3* tRCC) with up-regulated CircMET was employed to investigate its mechanism in cancer progression and post-transcriptional regulation.

**Methods:**

FISH and real-time PCR were performed to explore the expression and localization circMET in *NONO-TFE3* tRCC tissues and cells. The functions of circMET in tRCC were investigated by proliferation analysis, EdU staining, colony and sphere formation assay. The regulatory mechanisms among circMET, CDKN2A and SMAD3 were investigated by luciferase assay, RNA immunoprecipitation, RNA pulldown and targeted RNA demethylation system.

**Results:**

The expression of circMET was upregulated by NONO-TFE3 fusion in *NONO-TFE3* tRCC tissues and cells, and overexpression of circMET significantly promoted the growth of *NONO-TFE3* tRCC. Mechanistic studies revealed that circMET was delivered to cytosol by YTHDC1 in *N*^6^-methyladenosine (m^6^A)-depend manner. CircMET enhances mRNA decay of CDKN2A by direct interaction and recruitment of YTHDF2. Meanwhile, circMET competitively absorbed miR-1197 and prevented those from SMAD3 mRNA.

**Conclusions:**

CircMET promotes the development of *NONO-TFE3* tRCC, and the regulation to both CDKN2A and SMAD3 of circMET was revealed. CircMET has the potential to serve as a novel target for the molecular therapy of *NONO-TFE3* tRCC as well as the other cancer with high-expressing circMET.

**Supplementary Information:**

The online version contains supplementary material available at 10.1186/s12943-022-01497-w.

## Background

Xp11.2 translocation renal cell carcinoma (Xp11.2 tRCC), as an independent subset of RCC in the 2016 WHO classification [1], is characterized with poor prognosis of patients caused by high expression of transcript factor E3 (*TFE3*) fusion gene. Some strictly expressed genes, including *ASPL*, *PRCC*, *NONO* and *CLTC*, fused to *TFE3* gene [2–4], which result in the expression of fusion proteins with constitutive activity that become oncogenic drivers. The *NONO-TFE3* fusion gene, which retains 1 ~ 7 exons of *NONO* and 6 ~ 10 exons of *TFE3 *[5], remains the DNA-binding capacity by bHLH domain. Therefore, an increasing number of researches suggest that these high-expressed TFE3-fusion proteins can function as a major driver of cancer by regulating directly or indirectly downstream target genes. The NONO-TFE3 fusion maintains original functions of wild type TFE3, such as upregulation of lysosomal biogenesis genes via binding to promoter region [6]. Beyond that, NONO-TFE3 fusion regulates mitochondrial biosynthesis and metabolism through upregulation of nuclear respiratory factor 1 [7] and hypoxic adaptation via inducing the expression of hypoxia inducible factor 1 subunit alpha [8], which facilitate tumor progression of *NONO-TFE3* tRCC.

Circular RNAs (circRNAs), as a class of functional non-coding RNAs (ncRNAs), have a circular configuration formed by precursor mRNA back-splicing or skipping events without 5’ caps and 3’ poly(A) tail [9]. CircRNAs are resistant to exonuclease because of the circular structure, so they have a longer half-life than linear RNAs [10]. Although circRNAs is defined as a class of rubbish during the splicing process since it was firstly discovered, nowadays it has been considered as a pivotal regulator to participate in diverse physiological and pathological processes [11]. Mounting evidence shows that circRNAs play vital roles in carcinogenesis and cancer progression [12–15]. For example, circRNAs could sponge miRNAs to regulate expression of target gene post-transcriptionally, such as circAKT3 [16] and circSDHC [17] in RCC. Besides, circRNAs could directly bind to target protein and mediate the subcellular localization and degradation of protein [18, 19]. However, the potential molecular mechanism of circRNAs associated with the oncogenesis of Xp11.2 tRCC is still unclear.

Previous studies have verified that NONO-TFE3 fusion inhibits the expression of TRAF3IP2 antisense RNA 1 through directly binding to promoter region to promote the progression of *NONO-TFE3* tRCC [20]. Beyond this point, the chromatin immunoprecipitation sequencing (ChIP-seq) data reveal that NONO-TFE3 can also bind to promoter region of *MET *[6]. As a well-known and famous oncogene, MET plays an important role in cancer etiology, and there have been a few studies that have examined the high protein level of MET in Xp11.2 tRCC [21], including *NONO-TFE3* tRCC. Surprisingly, circRNAs from *MET* gene showed the pro-oncogenic effect and tumorigenicity strongly. Hsa_circ_0082003, one of circRNAs derived from *MET* gene, promotes the progression of cancer by sponging miR-145-5p in non-small-cell lung cancer [22], and circMET (also known as hsa_circ_0082002) induces hepatocellular carcinoma development and immune tolerance via miR-30-5p [23]. Although these results reveal the function of circRNAs derived from *MET* in tumor, the specific biology function of these circRNAs in Xp11.2 tRCC and the more intricate regulatory mechanisms is not fully revealed. Therefore, the role and mechanisms of circMET in Xp11.2 tRCC need to be addressed, which may provide therapeutic and prognostic value for Xp11.2 tRCC.

Herein, we found that circMET was upregulated by NONO-TFE3 fusion protein and silence of circMET could inhibit cell proliferation of *NONO-TFE3* tRCC*.* Mechanistically, we found that YTH domain-containing protein 1 (YTHDC1) promotes cytoplasmic export of circMET via binding to *N*^6^-methyladenosine (m^6^A) modification. CircMET directly interacted with cyclin dependent kinase inhibitor 2A (CDKN2A) mRNA through AAUAAA motif to promote the mRNA decay by YTH N6-methyladenosine RNA binding protein 2 (YTHDF2)-depend manner. In addition, circMET could function as a biological sponge to absorb miR-1197 and modulate the expression of SMAD family member 3 (SMAD3) post-transcriptionally. Therefore, our results indicate that circMET plays a critical role in cell proliferation of *NONO-TFE3* tRCC, which highlights a novel regulatory mechanism underlying tumor development.

## Materials and Methods

### Cell culture

HEK293T, HK-2, ACHN and 786-O cell lines were purchased from Type Culture Collection of Chinese Academy of Sciences (Shanghai, China), and the Xp11.2 tRCC cell lines UOK120 and UOK109 were gifts from Dr. W. Marston Linehan (National Cancer Institute, Bethesda, MD). The UOK120 (*PRCC-TFE3* fusion) and UOK109 (*NONO-TFE3* fusion) cell lines were derived from primary papillary cell carcinoma as described. Cells were cultured in complete media, including 89% DMEM/high glucose (Gibco, Grand Island, NY), 10% FBS (Gibco) and 1% penicillin–streptomycin (Gibco). Cells were cultured in 5% CO_2_ at 37 °C.

### Tissue samples

Patient samples were collected by Nanjing Drum Tower Hospital and confirmed by a senior pathologist (Department of Pathology, Nanjing Drum Tower Hospital). All patients provided informed consent that their tissues will be used into scientific research.

### RNA isolation and quantitative real-time PCR (real-time PCR) assays

Total RNA was isolated using Trizol reagent (Invitrogen) according to the product description. Cytoplasmic and nuclear RNA was extracted using an RNA Purification Kit (Norgen Biotek, Thorold, ON, Canada) as previously described. RNA was reverse-transcribed into cDNA and quantified by real-time PCR and the data were acquired with ChamQ Universal SYBR qPCR Master Mix (Vazyme Biotech Co.,Ltd, Nanjing, China). 18s rRNA was used for loading control. The level of 18s rRNA was also quantified to confirm the relative expression of circMET. The expression level of miRNAs was detected by miRNA Universal SYBR qPCR Master Mix (Vazyme). Single-stranded cDNA was synthesized as previously described. The U6 snRNA was chosen as internal control for normalization. The primers for RNAs were shown in Table S1.

### ChIP assay and dCas9-ChIP assay

ChIP assay and dCas9-ChIP assay were performed according to the protocol of a Pierce™ Agarose ChIP Kit (Thermo Scientific, Carlsbad, CA) to assess binding ability of NONO-TFE3 to *MET* promoter. Briefly, the cells were fixed, lysed and sonicated to appropriate fragments, which between 200 and 1200 bp. The prepared chromatin was precipitated overnight with specific antibodies or isotype-matched control IgG. Then, the immunoprecipitated chromatin was washed, eluted, purified as described above, and analyzed by real-time PCR. For dCas9-ChIP assay, UOK109 was co-transfected with dCas9 labeled with Flag and guide RNA targeted to *MET* promoter. While the dCas9 complex contained *MET* promoter fragment was enriched by anti-Flag, the proteins binding to *MET* promoter were enriched by dCas9 and then be detected [20, 24]. Primer sets targeting those regions containing potential NONO-TFE3 binding sites in *MET* promoter were provided in Table S2.

### Dual-luciferase reporter assay

HEK293T cells were transfected with indicated luciferase reporter plasmid together with pRL-TK and appropriate plasmids. Firefly and Renilla luciferase activities were measured using the Dual Luciferase Reporter Assay Kit (Vazyme), and Renilla luciferase activity was normalized to firefly luciferase activity.

### RNA immunoprecipitation (RIP) assay

RIP assays were performed according to the instructions of a Millipore Magna RIP Kit (Millipore, Darmstadt, Germany). The indicated cells were lysed with RIP lysis buffer containing protease and RNase inhibitor. After the cell lysate was cleared by centrifugation, the supernatant was inoculated with anti-AGO2 / YTHDF1 / YTHDF2 / YTHDC1 / IgG antibody-conjugated beads overnight at 4 °C. Then, the binding complexes were thoroughly washed, eluted, purified and analyzed by real-time PCR.

### MS2-RIP assay

MS2-RIP assays were performed according to the instructions of a Millipore Magna RIP Kit (Millipore, Darmstadt, Germany). The 6 × MS2 stem loop was fused to the circMET plasmid, and 12 × MS2 stem loop was added to the end of the CDKN2A mRNA 3’-UTR. The indicated cells were co-transfected with the target RNA expression vector fused with MS2 stem loop and the MS2-GFP vector, and these cells were lysed with RIP lysis buffer containing protease and RNase inhibitor. After the cell lysate was cleared by centrifugation, the supernatant was inoculated with anti-GFP/IgG antibody-conjugated beads overnight at 4 °C. Then, the binding complexes were thoroughly washed, eluted, purified and analyzed by real-time PCR or western blot.

### RNA pulldown assays

Biotinylated miR-1197 and circMET probe (Bersin Biotechnology Co., Ltd., Guangzhou, China) pulldown assays were performed according to the instructions. In brief, cells were lysed and sonicated. The probe was incubated with beads to generate probe-coated beads. The cell lysates were incubated with the probe-coated beads mixture at 4 °C overnight. After washing with the wash buffer, the RNA complexes bound to the beads were eluted and purified for further analysis.

### m^6^A RIP assay and circRNA-specific m^6^A RIP assay

Magna MeRIP™ m^6^A kit was chosen to assess m^6^A modification levels in target mRNA according to the manufacturer’s instructions (Millipore). Briefly, after saving 10% of the total RNA as input, the remaining RNAs were performed for immunoprecipitation with m^6^A antibody coated on magnetic beads A/G. Then, the binding complexes were thoroughly washed, eluted, purified and analyzed by real-time PCR. The same work flow was undertaken for experiments with circRNA-specific m^6^A RIP assay, except that the cell lysis was treated with RNase R to eliminate linear RNA before immunoprecipitation step.

### Western blot

Total protein was isolated from cells following various treatments. Cells were lysed in ice cold extraction buffer. After centrifuged, soluble fractions were mixed with 5 × loading buffer and heated. Proteins were separated using SDS-PAGE and transferred to PVDF membrane (Roche, Basel, Switzerland). Blots were blocked for 1 h at room temperature in 5% nonfat milk. Primary antibodies were incubated overnight at 4 °C in 3% BSA (Sigma Aldrich). HRP-conjugated secondary antibodies were incubated 1 h at room temperature. Signals were detected using chemiluminescent ECL reagent (Millipore), and band intensities were quantified using Image J software (National Institutes of Health). Additionally, ACTB was chosen as internal control.

### dCas13-EGFP labeling assay

To visualize circMET in cells, the dCas13-3 × EGFP was applied to show the location of endogenous circMET [25], and the guide RNA was designed to target to circMET (gRNA-circMET). Cells transfected with dCas13-3 × EGFP, gRNA-circMET and YTHDC1 were cultured on glass-bottomed dishes. Cells were washed once with PBS and fixed, then DAPI was used for nuclear stain. Images were acquired on a FV3000 confocal fluorescence microscope.

### Flow cytometry

Flow cytometry was performed according to the manufacture’s protocol. For cell cycle analysis, cells were incubated with reagents from a PI / RNase staining kit (BD Biosciences) and analyzed using a BD Beckman cytometer (BD Biosciences). Then, the data was analyzed by FlowJo software.

### CCK8, 5-Ethyny-2’-deoxyuridine (EdU) assay, clone forming and sphere formation

Cell proliferation assay was performed using the Cell Counting Kit 8 (CCK8; Vazyme). EdU (Beyotime, Shanghai, China) was operated according to the manufacture’s protocol. Transfected cells were seed into 6-well plate with 500 cells / well for 10 ~ 14 days to assess the clone-forming capacity. For sphere formation assay, proper cells were seeded in Ultra Low Attachment 96-well plates and cultured in DMEM supplemented with 10% FBS. The sphere pictures were taken 2 weeks later. Ultra-low attachment plates (cat. no. 174925) were purchased from Corning Incorporated (Corning, NY).

### Fluorescence in situ hybridization (FISH)

Cy3-labeled circMET probes were synthesized by GenePharma Technology (Shanghai, China). FISH was performed using a FISH kit (GenePharma) according to the manufacturer’s instructions. Nuclei were stained with DAPI. Images were acquired on a FV3000 confocal fluorescence microscope. The sequences are provided in Table S3.

### Targeted RNA demethylation system

Targeted RNA demethylation system was constructed by standard procedures including enzyme digestions, PCR and subcloning according to our previous study [20]. Briefly, the full length of ALKBH5 was fused to dCas13, and the NLS or NES was added to control the subcellular localization dCas13-NLS-ALKBH5/ dCas13-NES-ALKBH5. The gRNAs were designed for targeting circMET or CDKN2A. Then, the dCas13-ALKBH5 fusion protein and gRNAs were co-transfected into cells.

### Plasmid construction, short hairpin RNA (shRNA), lentivirus and cell transfection

The circMET sequence was subcloned into pLCDH-ciR, and 6 × MS2 was fused in this plasmid for MS2-RIP assay. Lentivirus and shRNA were purchased by OBiO Technology (Shanghai, China). Cells were transfected with plasmids using LipoFiter 3.0 (Hanbio, Shanghai, China) according to the manufacturer’s instructions. Treatments were administered 24 h after transfection. Cell were harvested 48 h after transfection. The sequences are provided in Table S4-S5.

### Animal experiment

The 6-week-old BALB/c nude mice were chosen for xenografts experiments and maintained in specific pathogen-free conditions. All procedures were approved by the Animal Care and Use Committee of Nanjing University under the animal protocol number SYXK (Su) 2009–0017. The mice were subcutaneously inoculated with 786-O cells stably transfected with lentiviruses carrying sh-NC/sh-circMET, respectively (5 × 10^6^, 200 uL). After 50 days, the mice were sacrificed. The tumor volume was calculated in the accordance with the formula (volume (cm^3^) = [width^2^ (cm^2^) × length (cm)]/2).

### Statistical analysis

Statistical analyses were performed using SPSS 22.0 software (SPSS Inc., Chicago, IL). GraphPad Prism 8.0 (GraphPad Software, San Diego, CA) was applied to plot the data. Student’s *t*-test and one-way analysis of variance (*ANOVA*) were used to assess the significance of differences. *P* < 0.05 was considered statistically significance (*P < 0.05, ** *P* < 0.01, and *** *P* < 0.001). All values are expressed as the means ± standard deviation.

## Results

### CircMET expression is significantly increased in *NONO-TFE3* tRCC

**Fig. 1:**
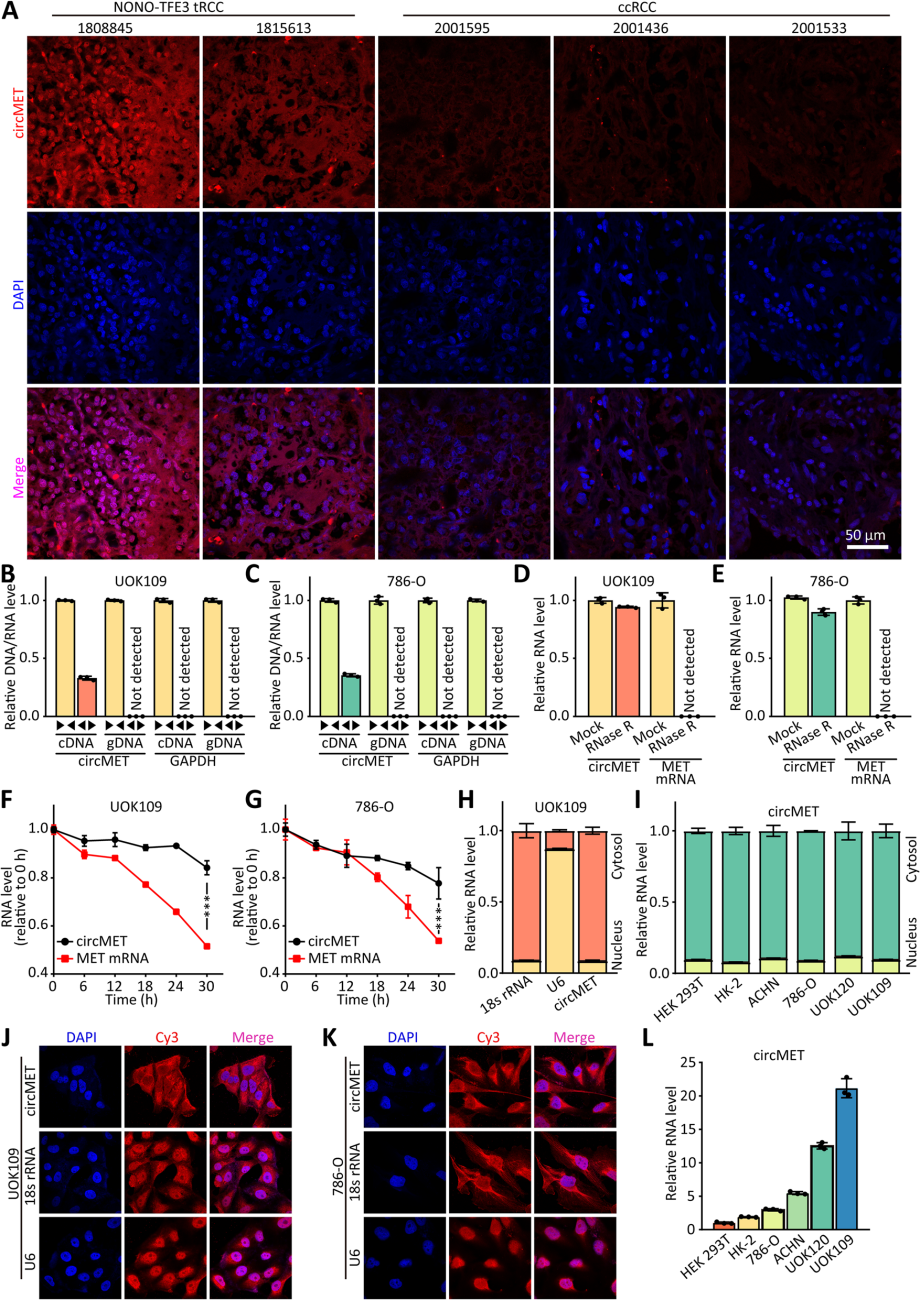
CircMET is significantly high-expressed in *NONO-TFE3* tRCC. **(A)** The RNA level of circMET (red) in *NONO-TFE3* tRCC and ccRCC was detected by FISH. Nuclei were visualized using DAPI (blue) counterstain. **(B-C)** cDNA and genomic DNA (gDNA) of UOK109 cells and 786-O cells were used to amplify circMET and GAPDH with divergent primers and convergent primers, respectively. **(D-E)** The RNA levels of circMET and MET mRNA in both UOK109 cells and 786-O cells were detected by real-time PCR after treatment of RNase R. **(F-G)** The expression of circMET and MET mRNA in both UOK109 cells and 786-O cells was detected by real-time PCR after treatment with α-amanitin at the indicated time points. **(H-I)** The subcellular distribution of circMET was analyzed via real-time PCR in ccRCC cell line (786-O and ACHN), tRCC cell lines (UOK109 and UOK120) and normal cell lines (HK-2 and HEK293T). U6 and 18s rRNA were used as nuclear and cytoplasmic markers, respectively. **(J-K)** The location of circMET (red) in UOK109 and 786-O cells was determined by FISH assay. U6 and 18s rRNA were used as nuclear and cytoplasmic markers, respectively. DAPI-stained nuclei are blue. **(L)** The RNA level of circMET was analyzed by real-time PCR assay in HEK293T, HK-2, ACHN, 786-O, UOK120 and UOK109 cells. The data are presented as the mean ± SD

In our previous study, *MET* was identified as a potential target gene of NONO-TFE3 by ChIP-seq. To explore if the expression of circRNAs derived from *MET* gene is correlated with *NONO-TFE3* tRCC, real-time PCR was performed to determine the expression of them. The results showed high level expression of circMET in *NONO-TFE3* Fig. S1A, B), and fluorescence in situ hybridization (FISH) was performed to confirm it (Fig. 1A). Notably, a positive correlation between circMET and MET was observed in clinical samples (Fig. S1C). There was also correlation between high circMET and higher tumor stage (Fig. S1D).

To verify that circMET is produced by the head to tail splicing, the convergent and divergent primers were used to amplify circMET and linear MET, respectively. Real-time PCR revealed that circMET could only be detected in cDNA but not in gDNA (Fig. 1B, C), whereas linear MET was amplified from both cDNA and gDNA by the convergent primers. Moreover, we further showed that circMET transcripts was more stable than linear MET in response to RNase R through real-time PCR (Fig. 1D, E). Similarly, after treatment with α-amanitin, an inhibitor of transcription, the result showed that circMET was resistant at the indicated time points, whereas linear MET mRNA transcripts were rapidly degraded (Fig. 1F, G), indicating that circMET is more stable in 786-O and UOK109 cells. In addition, the sequence of circMET consisting of one exon had a length of 1214 nucleotides (Fig. S1E). Next, the subcellular localization of circMET was observed with FISH and nuclear/cytoplasmic fractionation assays. The results showed that circMET was predominantly located in the cytosol (Fig. 1H-K).

To investigate the role of circMET in *NONO-TFE3* tRCC progression, we first evaluated circMET expression levels in various RCC cell lines and normal cell. The lowest expression of circMET was observed in HEK293T cell line, whereas the highest level was detected in UOK109 cell line which was derived from tumor tissue of patient with *NONO-TFE3* tRCC (Fig. 1L).

### CircMET accelerates proliferation of *NONO-TFE3* tRCC

**Fig. 2:**
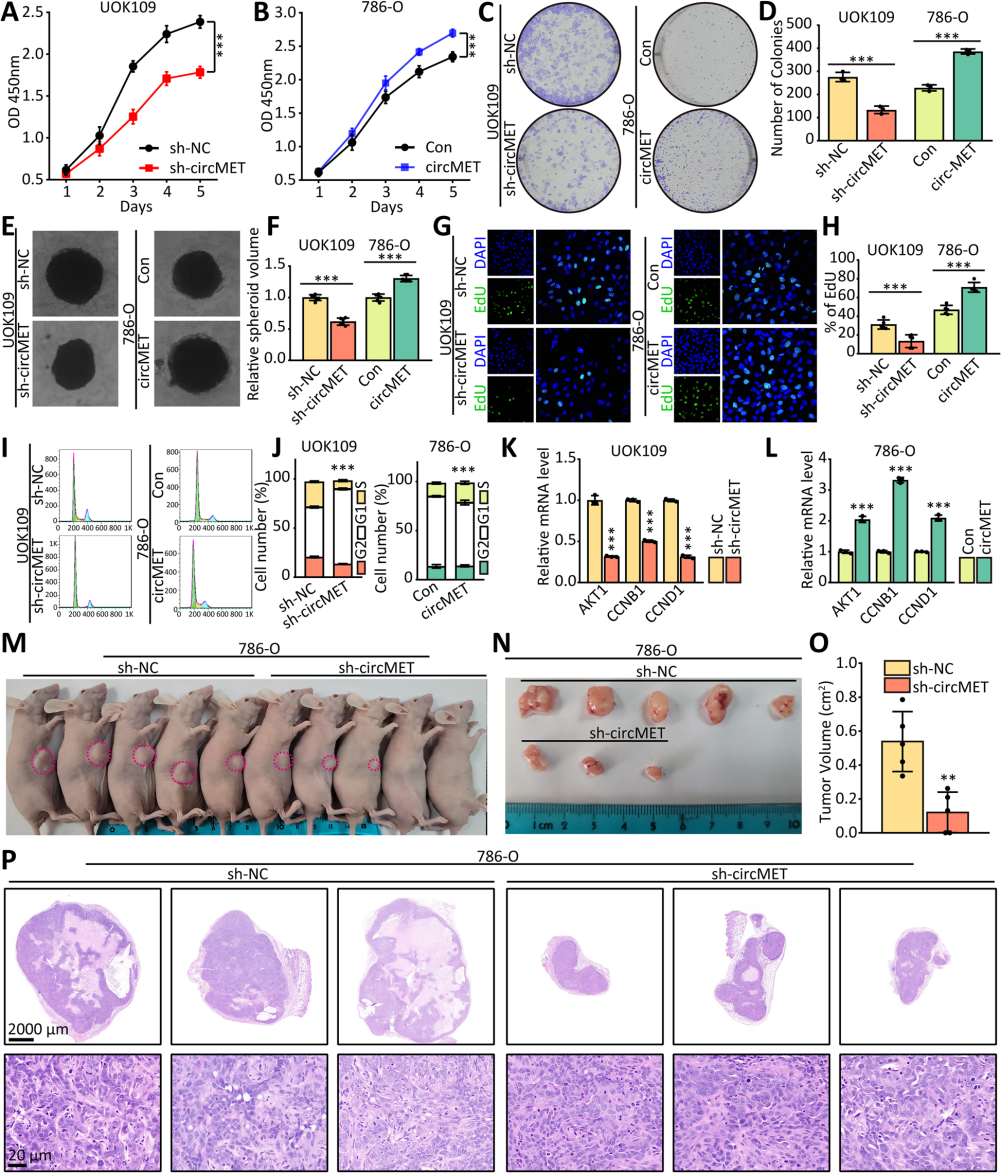
High-expressed circMET promotes the proliferation of *NONO-TFE3* tRCC. **(A-F)** The effects of circMET knockdown or overexpression on the proliferation of UOK109 and 786-O cells respectively were examined by CCK-8 assay **(A-B)**, colony formation assays **(C-D)** and tumor sphere formation **(E–F)**. **(G-H)** EdU assays were used to detect the proliferation rate of UOK109 and 786-O cells after transfected with indicated lentivirus. **(I-J)** Cell cycle phases were analyzed using flow cytometry after transfected with indicated lentivirus. **(K-L)** circMET mediates the expression of genes related with cell proliferation and cell cycle in UOK109 and 786-O cells. **(M–N)** Nude mice were injected subcutaneously with 786-O cells and tumor formation monitored over a period of several weeks. **(O)** The tumor volume was measured as indicated. **(P)** Representative H&E staining of xenograft tumors. The data are presented as the mean ± SD, ***P < 0.001

To determine the functional role of circMET in biological behavior of *NONO-TFE3* tRCC, the effects of downregulated or upregulated circMET on cancer cell growth was investigated. ShRNA was designed to diminish expression of circMET in UOK109 cells (Fig. S2A), and lentivirus was applied to up-regulate circMET in 786-O cells (Fig. S2B). The results showed that cell proliferation (Fig. 2A, B), colony formation (Fig. 2C, D) and tumor sphere formation (Fig. 2E, F) were inhibited by knockdown of circMET compared with the negative control in UOK109 cells. Moreover, circMET overexpression significantly increased cell proliferation, colony formation and tumor sphere formation in 786-O cell line.

Additionally, using an EdU assay, we confirmed that slicence of circMET reduced the rate of DNA replication in UOK109 cells, and opposite results were observed in 786-O cells with ectopic expression of circMET (Fig. 2G, H). Meanwhile, the result of cell cycle assay showed that silence of circMET resulted in a decrease in the S-phase population, with a concomitant increase in the G1 population (Fig. 2I, J), and overexpression of circMET in 786-O cells lead to an increase in the number of cells in S-phase. The mRNA levels of regulating proteins (AKT1, CCNB1 and CCND1) was used to confirm these results (Fig. 2K, L). Additionally, dramatically reduced tumor size was observed in the mice injected with 786-O-sh-circMET cells as compared to the control group (Fig. 2M-P). These findings indicate that circMET might accelerate proliferation of *NONO-TFE3* tRCC.

### The transcription of circMET is enhanced by NONO-TFE3

**Fig. 3:**
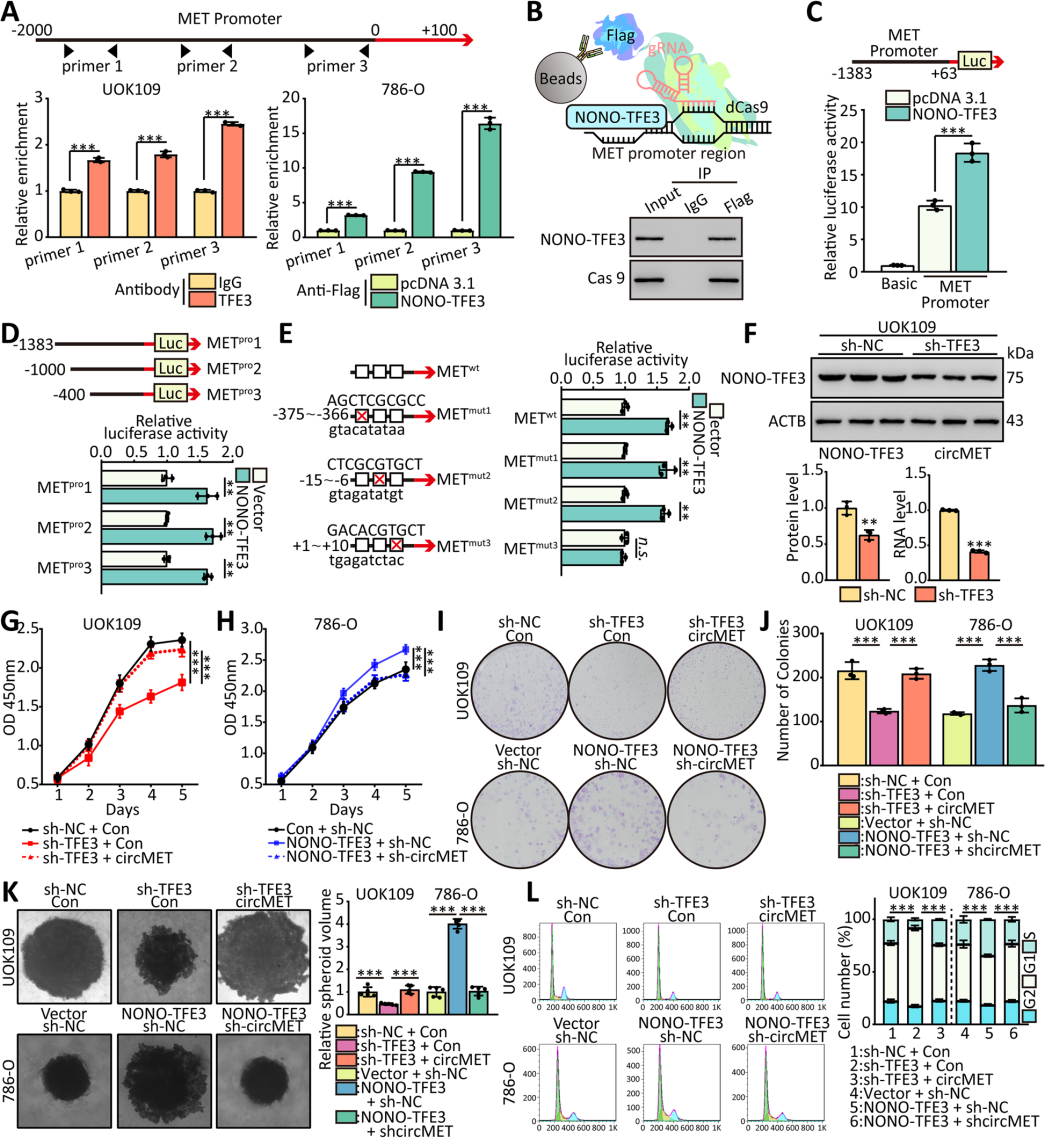
The transcription of circMET is enhanced by NONO-TFE3. **(A)** ChIP assay and real-time PCR were used to determine the binding affinity of NONO-TFE3 to *MET* promoter regions in UOK109 and 786-O cells. Real-time PCR with IgG was performed as the control. **(B)** Schematic summary of the dCas9-gRNA-guided ChIP (upper); Western blot was performed after dCas9-gRNA-guided ChIP (lower). **(C-D)** HEK293T cells were co-transfected with *MET* promoter-luciferase truncations and NONO-TFE3 plasmids, and the luciferase activity was determined using a dual luciferase reporter assay after 48 h. **(E)** Dual luciferase assay of HEK293T cells co-transfected with firefly luciferase constructs containing the wild-type or mutant NONO-TFE3 potential binding sites of *MET* promoter and NONO-TFE3 plasmids were performed. **(F)** The protein level of NONO-TFE3 and the circMET expression levels were detected after transfection with sh-TFE3 for 48 h. **(G-H)** Cell viability of UOK109 and 786-O cells was determined using CCK-8 assays after transfection for 48 h. **(I-K)** A colony formation and tumor sphere formation assay were used to determine the colony and tumor sphere formation ability of UOK109 and 786-O cells co-transfected with indicated lentivirus. **(L)** Cell cycle analysis was performed using flow cytometry in cells transfected with indicated lentivirus. The data are presented as the mean ± SD, *n.s.* = non-significant, **P < 0.01, ***P < 0.001

**Fig. 4:**
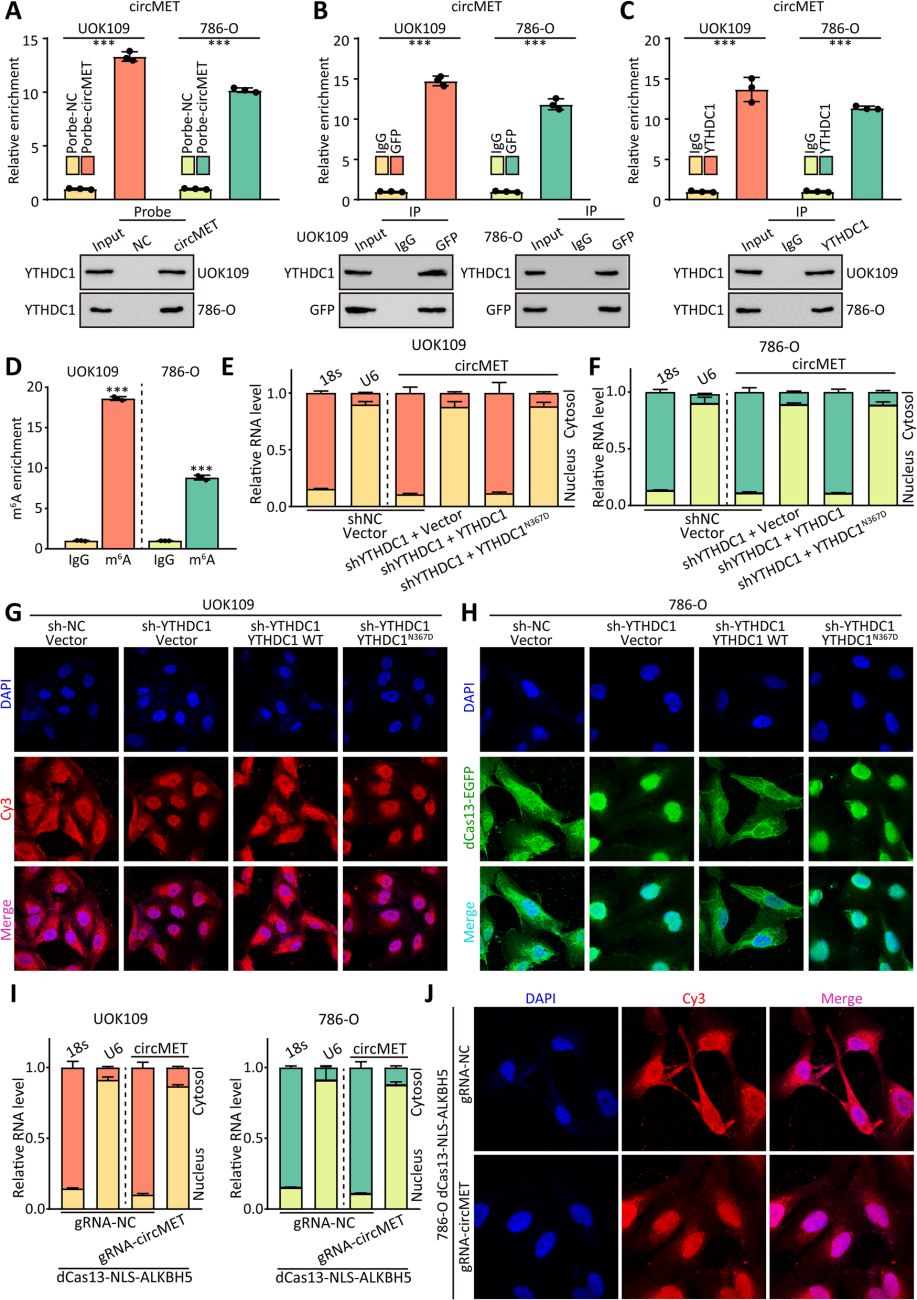
CircMET is exported to cytoplasm by YTHDC1 in m^6^A-depend manner. **(A)** The circMET-protein complex was pulled down by circMET junction probe with protein extracts from UOK109 and 786-O cells. The efficiency of circMET junction probe was detected by real-time PCR (upper), and the enrichment of YTHDC1 was detected by western blot (lower). **(B-C)** MS2-RIP and RIP assays were performed to confirm the association of YTHDC1 with circMET. The relative enrichment of circMET associated with YTHDC1 was detected by real-time PCR (upper), and IP efficiency of YTHDC1-antibody was showed in western blot (lower). IgG antibody served as a control. **(D)** Abundance of circMET among RNA immunoprecipitated with anti-m^6^A antibody in UOK109 and 786-O cells was measured by real-time PCR and normalized to IgG. **(E–F)** The subcellular distribution of circMET was analyzed via real-time PCR in UOK109 and 786-O cells after transfected with indicated lentivirus. U6 and 18s rRNA were used as nuclear and cytoplasmic markers, respectively. **(G)** The location of circMET (red) in UOK109 transfected with indicated lentivirus was determined by FISH assay. DAPI-stained nuclei are blue. **(H)** CircMET (green) in UOK109 transfected with indicated lentivirus was lighted by dCas13-3 × EGFP and gRNA targeted to circMET. DAPI-stained nuclei are blue. **(I)** The subcellular distribution of circMET was analyzed via real-time PCR in UOK109 and 786-O cells after transfected with indicated lentivirus. U6 and 18s rRNA were used as nuclear and cytoplasmic markers, respectively. **(J)** The location of circMET (red) in UOK109 transfected with indicated lentivirus was determined by FISH assay. DAPI-stained nuclei are blue. The data are presented as the mean ± SD, ***P < 0.001

To prove the hypothesis that NONO-TFE3 enhanced the transcription of circMET, generated from *MET* gene, we conducted ChIP assays and found that NONO-TFE3 directly interacted with the NONO-TFE3 binding sites within the *MET* promoter in UOK109 and 786-O cells (Fig. 3A). This finding was confirmed by the result of dCas9-gRNA-guided ChIP (Fig. 3B; Fig. S3). Furthermore, dual-luciferase reporter gene assay revealed that NONO-TFE3 fusion directly targeted the promoter of *MET* to positively regulate the luciferase activity (Fig. 3C). To further clarify the regulatory mechanism, three promoter regions, designated as pGL3-MET^pro^1 (− 1383 ~  + 48), pGL3- MET^pro^2 (− 1000 ~  + 48) and pGL3- MET^pro^3 (− 400 ~  + 48), were cloned into luciferase reporter plasmid to identify the binding sites of NONO-TFE3 fusion. Dual-luciferase reporter assays revealed that NONO-TFE3 fusion could bind to the region of − 400 ~  + 48 (Fig. 3D). Then, to further determine the exact binding sites, mutations was made at 3 putative binding sites, respectively. HEK293T cells were co-transfected with the NONO-TFE3/Vector and MET^WT^, MET^Mut1^, MET^Mut2^, or MET^Mut3^. The luciferase activity of both MET^Mut1^ and MET^Mut2^ showed no significant change, whereas MET^Mut3^ showed significantly change, indicating that the actual site of NONO-TFE3 binding to promoter region of *MET* was + 1 ~  + 10 (Fig. 3E). Next, we altered NONO-TFE3 protein levels by loss- and gain-of-functions in vitro and detected the expression of circMET in UOK109 and 786-O cells, respectively. The expression of circMET was attenuated by knockdown of NONO-TFE3 compared with the negative control in UOK109 cells (Fig. 3F), and similar results were shown in 786-O cells (Fig. 4A). Among these circRNAs derived from *MET* gene, circMET showed the most significant change after knockdown of NONO-TFE3 in UOK109 cells (Fig. 4B). These data demonstrate that NONO-TFE3 fusion can bind to MET and up-regulate the expression of circMET.

Since the expression of circMET was upregulated by the high expressed NONO-TFE3 fusion, to verify that NONO-TFE3 could promote tumor growth through circMET, a series of rescue experiments were performed. Cell proliferation, colony formation and tumor sphere formation were mitigated by silencing the expression of NONO-TFE3 compared with the negative control in UOK109 cells, but the overexpression of circMET reversed this phenomenon (Fig. 3G-K). Concordantly, overexpression of NONO-TFE3 significantly enhanced the capacities of cell proliferation, colony formation and tumor sphere formation in 786-O cell line. After transfected with circMET shRNA, the capacities of cell proliferation, colony formation and tumor sphere formation came back to control level. The data of flow cytometry analysis including cell cycle also confirmed the above results (Fig. 3L). These findings reveal that NONO-TFE3 promotes *NONO-TFE3* tRCC progression through up-regulating expression of circMET.

### CircMET is exported to cytoplasm by YTHDC1 in m^6^A-depend manner

To explore the potential molecular mechanisms of circMET in regulating *NONO-TFE3* tRCC, we applied the Encyclopedia of RNA Interactomes (ENCORI, http://starbase.sysu.edu.cn/) [26, 27] to predict the potential binding protein of circMET. Then, we analyzed each potential binding proteins, and select 17 proteins, which are involved in RNA export, for next research. After transfected with shRNAs of these proteins, sh-YTHDC1 caused cytoplasmic distribution of circMET strongly (Fig. S5A, B). After that, we performed RNA pulldown assays to clarify why circMET localized in the cytosol. The results of RNA pulldown assays indicated that YTHDC1 could enriched by circMET both in UOK109 and 786-O cells (Fig. 4A), and the result of MS2 RNA immunoprecipitation (MS2-RIP) assay indicated that exogenous circMET could bind to YTHDC1 (Fig. 4B). Moreover, the RIP assay showed that circMET was remarkably enriched by YTHDC1 antibody, which suggested that YTHDC1 could bind to circMET (Fig. 4C). As a member of m^6^A reader proteins, YTHDC1 is known to be involved in RNA splicing [28] and RNA nuclear export [29]. According to the analysis of circMET from WHISTLE (https://whistle-epitranscriptome.com/) [30]and m6AVar database (http://m6avar.renlab.org/) [31], we found that there are seven potential m^6^A modification site in circMET (Fig. S5C). The results of m^6^A RIP analyses showed that m^6^A was significantly enriched at circMET (Fig. 4D). Moreover, circRNA-specific m^6^A RIP was performed, the total RNA of UOK109 cells was treated with RNase R to degrade linear RNA before m^6^A RIP (Fig. S5D). The results of circRNA-specific m^6^A RIP analyses indicated that the 270^th^/816^th^/831^th^ nucleotide position showed higher probability for m^6^A modification (Fig. S5D, E). To further clarify the actual modified sites, four mutated circMET, designated as circMET^A270T^, circMET^A816T^, circMET^A831T^ and circMET^A270/831T^, were cloned into pLCDH-ciR plasmid. The UOK109 cells were transfected with wild-type circMET or these four circMET contained mutation, and the result of MeRIP assay and real-time showed that circMET^A270T^, circMET^A816T^ and circMET^A270/831T^ could abolish the m^6^A level of circMET (Fig. S5F). Besides, the circMET^A270T^, circMET^A816T^ and circMET^A270/831T^ could also abolish the interaction between circMET and YTHDC1 according to the results of RIP and MS2-RIP assays (Fig. S5G-I).

To verify that circMET was exported to cytosolic in the YTHDC1-depend manner, nuclear/cytosol fractions were isolated prior to RNA extraction. YTHDC1 knockdown caused the retention of circMET in nucleus (Fig. 4E, F), and enforced expression of YTHDC1 wild-type (WT), but not m^6^A-binding defective YTHDC1 (YTHDC1^N367D^) [32], rescued the defective cytoplasmic export of circMET, which is induced by depletion of YTHDC1. In order to further confirm the real-time PCR results, FISH assay was performed to confirm that YTHDC1 could promote the cytosolic localization of circMET (Fig. 4G). Furthermore, inactivated Cas13 (dCas13) fused to 3 × EGFP was applied to label the circMET in living cell co-transfected with guide RNA (gRNA) targeted to circMET [25], and the result was consistent with the concept above (Fig. 4H). Meanwhile, the overexpressed circMET^A270/831T^ was detained in the nucleus (Fig. S5J, K). To further confirm that YTHDC1 promoted the cytosolic localization of circMET via m^6^A modification, RNA demethylases alkB homolog 5 (ALKBH5) fused to dCas13 was used to remove the m^6^A modification on circMET, and nuclear localization signal (NLS) was added to this fusion protein for the nuclear localization of dCas13-NLS-ALKBH5 fusions [33]. Cytoplasmic distribution of circMET was diminished in UOK109 and 786-O cells transfected with dCas13-NLS-ALKBH5 and gRNA targeted to circMET (gRNA-circMET, Fig. 4I, J; Fig. S5L-O). On the other hand, the corresponding modified sites of MET mRNA could alter the protein level of MET and the cytoplasmic distribution of MET mRNA instead of mRNA level (Fig. S6).

To further explore the biological function of m^6^A modification of circMET in *NONO-TFE3* tRCC, CCK-8, colony formation and tumor sphere formation assays were performed (Fig. S7). The results revealed that cell proliferation and growth were inhibited after removing m^6^A modification of circMET compared with the negative control in UOK109 cells. Collectively, these data imply that YTHDC1 could bind to the m^6^A modification on the circMET, and promote the cytosolic localization of circMET via m^6^A modification.

### CircMET down‑regulates CDKN2A mRNA by direct binding

**Fig. 5:**
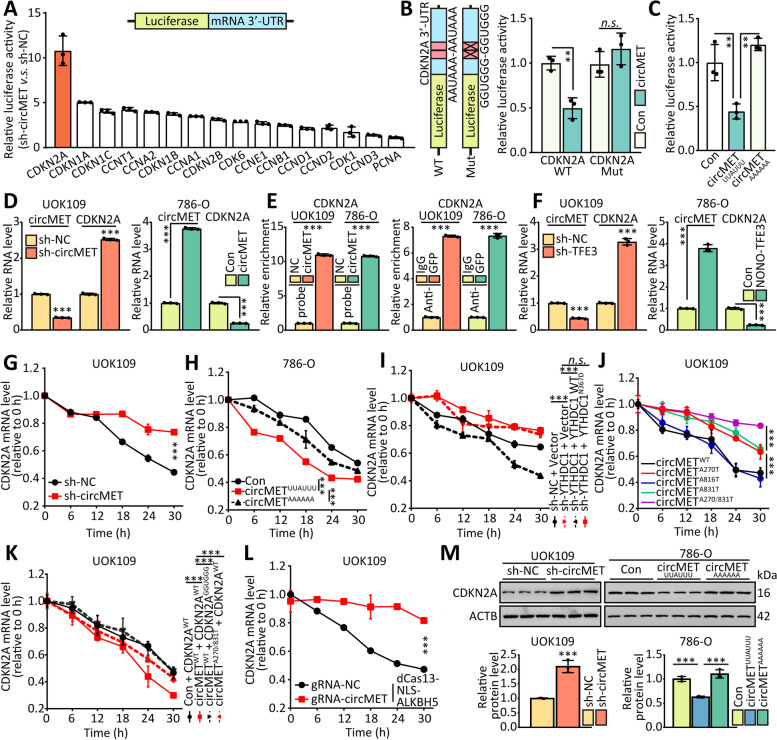
CircMET down‑regulates CDKN2A mRNA by direct binding. **(A)** HEK293T cells were co-transfected with indicated mRNA 3’-UTR luciferase truncations and sh-circMET, and the luciferase activity was determined using a dual luciferase reporter assay after 48 h. **(B)** Dual luciferase assay of HEK293T cells co-transfected with firefly luciferase constructs containing the wild-type or mutant AAUAAA motif in CDKN2A mRNA 3’-UTR and circMET plasma were performed. **(C)** Dual luciferase assay of HEK293T cells co-transfected with CDKN2A mRNA 3’-UTR luciferase constructs and wild-type or mutant circMET plasma were performed. **(D)** The RNA levels of circMET and CDKN2A mRNA in both UOK109 cells and 786-O cells were detected by real-time PCR after silence or overexpression of circMET. **(E)** RNA pulldown and MS2-RIP assays were performed to confirm the association of CDKN2A mRNA with circMET. **(F)** The RNA levels of circMET and CDKN2A mRNA in both UOK109 cells and 786-O cells were detected by real-time PCR after silence or overexpression of NONO-TFE3. **(G-L)** The stability of CDKN2A mRNA in cells transfected with indicated lentivirus after treatment with α-amanitin. **(M)** The protein level of CDKN2A in both UOK109 cells and 786-O cells were detected by western blot after silence or overexpression of circMET. The data are presented as the mean ± SD, *n.s.* = non-significant, **P < 0.01, ***P < 0.001

Since it has been established that circMET accelerates proliferation of NONO-TFE3 tRCC, we want to know whether circMET can regulate the expression of genes associated with cell cycle. AREsite analysis (http://rna.tbi.univie.ac.at/AREsite2) [34] and BLAST analysis suggested that the UUAUUU site inside of circMET might directly bind to the 3′-UTR of 16 genes, which regulate cell cycle, with AAUAAA motif. The 3’-UTR of these genes was cloned into luciferase reporter plasmid and co-transfected with circMET shRNA respectively, and the result of dual-luciferase assay indicated the most notably upregulated luciferase activity after silence of circMET was CDKN2A (Fig. 5A). CDKN2A, as a tumor suppressor gene, encodes the protein of p16, which could arrest cell cycle of cells [35–37]. Additionally, overexpression of circMET decreased the luciferase activity of CDKN2A 3’-UTR reporter, but showed no effort to the CDKN2A 3’-UTR reporter contained the mutation of AAUAAA motif (Fig. 5B). Likewise, this luciferase activity could not be affected by circMET contain mutation of UUAUUU site (circMET^AAAAAA^, Fig. 5C).

Then, the validation of real-time PCR showed that the expression of CDKN2A were increased in UOK109 cells upon circMET knockdown (Fig. 5D). Similarly, overexpression of circMET down-regulated the level of CDKN2A in 786-O cells. The pulldown assay showed that circMET could interact with CDKN2A mRNA directly, and the results of MS2-RIP revealed that CDKN2A mRNA was significantly enriched in circMET (Fig. 5E). In addition, the increased CDKN2A level was observed in UOK109 cells transfected with TFE3 shRNA (Fig. 5F), and ectopic NONO-TFE3 expression diminished the mRNA level of CDKN2A in 786-O cells.

Next, to test whether circMET regulates the stability of CDKN2A mRNA, we treated UOK109 and 786-O cells with α-amanitin to block RNA polymerase II-mediated new RNA synthesis and then measured the loss of CDKN2A and GAPDH over a 30-h period. Knockdown of circMET clearly elongated the half-life of CDKN2A mRNA (Fig. 5G; Fig**.** S8A), whereas ectopic overexpression of circMET, but not that of circMET with mutation, reduced the half-life of CDKN2A mRNA (Fig. 5H; Fig. S8B). Aside from this, the changes of YTHDC1 expression induced the corresponding alterations of CDKN2A mRNA stability (Fig. 5I; Fig. S8C), and the mutation of m^6^A modification could enhance the stability of CDKN2A mRNA (Fig. 5J; Fig. S8D). Moreover, the CDKN2A contained mutation of AAUAAA motif would not be influenced by circMET (Fig. 5K; Fig. S8E). Additionally, the half-life of CDKN2A mRNA was elongated after removing the m^6^A modification on circMET in UOK109 cells (Fig. 5J; Fig. S8F). The protein level of CDKN2A increased by circMET knockdown (Fig. 5K), and a restraint of CDKN2A protein level was led by ectopic expression of circMET without mutation in 786-O cells.

To confirm that circMET could promote tumor growth through reducing CDKN2A mRNA stability, a series of rescue experiments were performed. Cell proliferation, colony formation and tumor sphere formation were inhibited by silencing the expression of circMET compared with the negative control in UOK109 cells, but the knockdown of CDKN2A reversed this phenomenon (Fig. S9A-C). Concordantly, overexpression of circMET significantly enhanced the capacities of cell proliferation, colony formation and tumor sphere formation in 786-O cell line. After overexpression of CDKN2A, the capacities of cell proliferation, colony formation and tumor sphere formation came back to control level. The data of cell cycle also confirmed the above results (Fig. S9D). Taken together, circMET could bind to CDKN2A mRNA and reduce the half-life of CDKN2A mRNA.

### CircMET accelerates the decay of CDKN2A mRNA by recruitment of YTHDF2

**Fig. 6:**
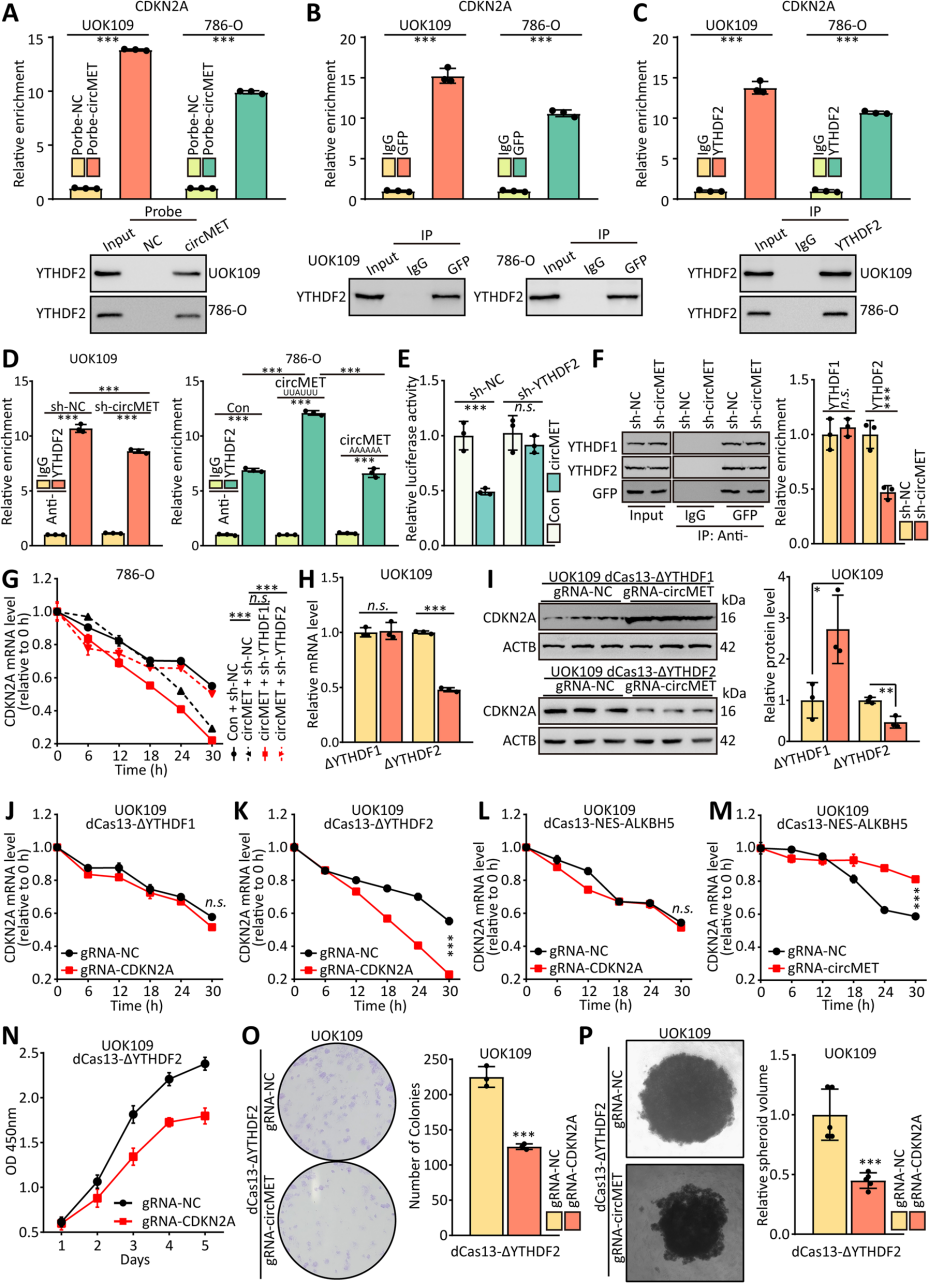
CircMET accelerates the decay of CDKN2A mRNA by recruitment of YTHDF2. **(A)** The circMET-protein complex was pulled down by circMET junction probe with protein extracts from UOK109 and 786-O cells. The enrichment of CDKN2A mRNA was detected by real-time PCR (upper), and the enrichment of YTHDF2 was detected by western blot (lower). **(B-C)** MS2-RIP and RIP assays were performed to confirm the association of YTHDF2 with CDKN2A mRNA. The relative enrichment of CDKN2A mRNA associated with YTHDF2 was detected by real-time PCR (upper), and IP efficiency of YTHDF2/GFP-antibody was showed in western blot (lower). IgG antibody served as a control. **(D)** RIP assay was performed with UOK109 and 786-O cells transfected indicated lentivirus, and the relative enrichment of CDKN2A mRNA associated with YTHDF2 was detected by real-time PCR. IgG antibody served as a control. **(E)** Luciferase activity of CDKN2A-3’-UTR was measured after co-transfected with circMET and sh-YTHDF2. **(F)** Abundance of YTHDF1/2 among MS2-RIP with anti-GFP antibody from cells transfected with indicated plasmid was measured by western blot. **(G)** The stability of CDKN2A mRNA in 786-O cells transfected with indicated lentivirus after treatment with α-amanitin. **(H-I)** The mRNA and protein level of CDKN2A were measured after transfected with indicated gRNA and dCas13 fusions. **(J-M)** The stability of CDKN2A mRNA in cells transfected with indicated lentivirus after treatment with α-amanitin. **(N)** Cell viability of UOK109 and 786-O cells was determined using CCK-8 assays after transfection for 48 h. **(O-P)** A colony formation and tumor sphere formation assay were used to determine the colony and tumor sphere formation ability of UOK109 and 786-O cells co-transfected with indicated lentivirus. The data are presented as the mean ± SD, *n.s.* = non-significant, *P < 0.05, **P < 0.01, ***P < 0.001

Because circMET and m^6^A methylation appeared to reduce the expression of CDKN2A, we predicted the potential binding proteins of circMET by ENCORI and designed shRNAs of them. The CDKN2A 3’-UTR luciferase reporter plasmid and these shRNAs were co-transfected into HEK293T cells, respectively, and the result of dual-luciferase assay indicated the more notably upregulated luciferase activity of CDKN2A were sh-HNRNPD, sh-YTHDF2, sh-TARDBP and sh-RC3H1 among the 90 shRNAs (Fig. S10A). According to the result of MS2-RIP based on circMET contacted MS2 stem loop, the HNRNPD, YTHDF2, TARDBP and RC3H1 could interact with circMET (Fig. S10B). Then, the result of MS2-RIP based on CDKN2A mRNA contacted MS2 stem loop suggested that the interaction between CDKN2A mRNA and YTHDF2 could be weakened by sh-circMET (Fig. S10C, D). Thus, we speculated that circMET recruit YTHDF2 to CDKN2A transcript. As assessed by pulldown, MS2-RIP and RIP analyses, YTHDF2 interacted strongly with circMET (Fig. 6A-C). Moreover, the result of RIP assay showed that CDKN2A mRNA was enriched in YTHDF2 complex, and circMET shRNA decreased the enrichment of CDKN2A mRNA. Overexpression of wild type circMET, but not mutated circMET, enhanced the enrichment of CDKN2A mRNA in YTHDF2 complex (Fig. 6D). The luciferase reporter assay showed that overexpressed circMET downregulated the luciferase activity of CDKN2A 3’-UTR (Fig. 6E), but the high level of circMET showed no influence after silence of YTHDF2. The MS2-RIP/western blot analysis indicated that YTHDF2 interacted strongly with CDKN2A mRNA, not YTHDF1 (Fig. 6F).

Consistent with the findings reported above, the decreased decay rates of CDKN2A mRNA were nearly restored back to normal by knockdown of YTHDF2 in UOK109 cells (Fig. 6G; Fig. S11A, B). Targeted m^6^A read system was applied to confirm the above results [20]. YTHDF2^1–400^ (ΔYTHDF2) decreased the protein and mRNA level of CDKN2A in UOK109 cells transfected with gRNA-circMET, and YTHDF1^1–350^ (ΔYTHDF1) increased the protein level of CDKN2A (Fig. 6H, I). Consistent with prediction, ΔYTHDF1 shown no significant impact in the mRNA level of CDKN2A in UOK109 cells transfected with gRNA-circMET. Correspondingly, ΔYTHDF2, but not ΔYTHDF1, and gRNA-circMET accelerated the mRNA decay of CDKN2A (Fig. 6J, K; Fig. S11C, D). Interestingly, removing the m^6^A modification of CDKN2A mRNA located in the cytosol, using gRNA-CDKN2A and dCas13-ALKBH5 labeled with nuclear export signal (NES) [33], cannot change the half-time of CDKN2A mRNA (Fig. 6L; Fig. S11E, F), and removing the m^6^A modification of circMET located in the cytosol enhanced the stability of CDKN2A mRNA (Fig. 6M; Fig. S11G, H), indicating that YTHDF2 might promote decay of CDKN2A mRNA by interacting with the m^6^A modification on circMET, but not on CDKN2A mRNA. As a further test of this hypothesis, the MS2-RIP and RNA pulldown assays were performed. The results showed that removing the m^6^A modification of CDKN2A mRNA located in the cytosol could not change the interaction between CDKN2A mRNA and YTHDF2, but removing the m^6^A modification of circMET located in the cytosol could do it (Fig. S11I-L).

CCK-8, colony formation and tumor sphere formation assays were performed to illuminate the impact of YTHDF2 to the behavior of UOK109 cells. The results revealed that cell proliferation and growth were inhibited by transfection with ΔYTHDF2 and gRNA-CDKN2A compared with the negative control in UOK109 cells (Fig. 6N-P). Collectively, YTHDF2 mediates the proliferation of *NONO-TFE3* tRCC by recognizing circMET and facilitating the CDKN2A mRNA decay.

### CircMET functions as a ceRNA to sponge miRNAs

**Fig. 7:**
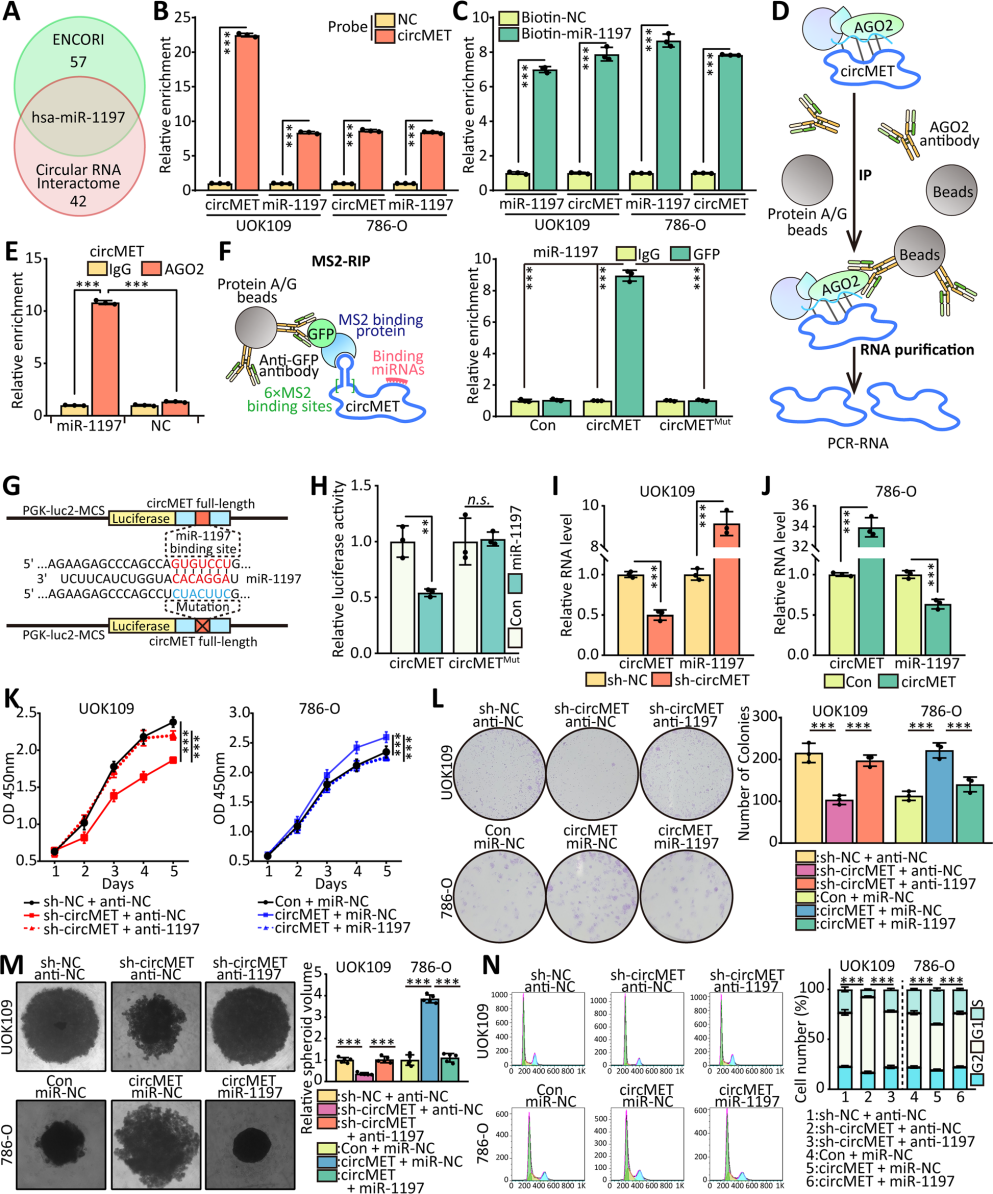
CircMET functions as a ceRNA to sponge miRNAs. **(A)** Schematic of the selection for the direct downstream target of circMET. **(B-C)** The circMET-protein complex and miR-1197-Biotin complex were enriched by circMET junction probe or Biotin-antibody with protein extracts from UOK109 and 786-O cells. The enrichment of circMET and miR-1197 was detected by real-time PCR. **(D)** Model of AGO2-RIP assay. **(E)** RIP assays were performed using AGO2 antibody in UOK109 cells, then the enrichment of circMET was detected by real-time PCR. **(F)** Model of MS2-RIP assay (left). MS2-RIP-derived RNA was examined by real-time PCR (right). The levels of the real-time PCR products were normalized relative to IgG control. **(G)** Schematic illustration of circMET wild type and mutation luciferase reporter vectors. **(H)** HEK293T cells were co-transfected with miRNA mimics and wild-type or mutant circMET luciferase reporter vector, and luciferase reporter activity was detected. **(I-J)** The effect of circMET on miR-1197 expression in UOK109 cells was analyzed by real-time PCR after overexpression or knockdown of circMET. **(K)** Cell viability of UOK109 and 786-O cells was determined using CCK-8 assays after transfection for 48 h. **(L-M)** A colony formation and tumor sphere formation assay were used to determine the colony and tumor sphere formation ability of UOK109 and 786-O cells co-transfected with indicated lentivirus. **(N)** Cell cycle analysis was performed using flow cytometry in cells transfected with indicated lentivirus. The data are presented as the mean ± SD, *n.s.* = non-significant, **P < 0.01, ***P < 0.001

Numerous studies have confirmed that circRNAs exert critical roles in regulating gene expression by function as a biological sponge for miRNAs. The intracellular distribution of circMET suggested that circMET might also have a post-transcriptional regulation function that contributes to the proliferation of *NONO-TFE3* tRCC. Then, we predicted potential miRNA targeting sites on circMET using ENCORI and Circular RNA Interactome (https://circinteractome.nia.nih.gov/) [38] database and screened out only one candidate miRNA, miR-1197 (Fig. 7A). To determine the interaction between circMET and the listed miR-1197, we performed pulldown assays and found that miR-1197 could be enriched by probe of circMET (Fig. 7B), and circMET was enriched by biotin-labeled miR-1197 (Fig. 7C). The RIP assay showed that circMET could interact with AGO2 directly, which has been identified as a member of RNA-induced silencing complex (RISC) [39], while miR-1197 were up-regulated (Fig. 7D, E). Moreover, the results of MS2-RIP revealed that miR-1197 was significantly enriched in circMET compared with circMET contained mutation of potential miR-1197 binding site (Fig. 7F).

Dual-luciferase assay was processed to confirm the interaction between miR-1197 and circMET. Luciferase activities were repressed by co-transfection with miR-1197 compared with the control (Fig. 7G, H). However, this inhibitory effect was diminished by mutation of the putative miR-1197 binding site in the circMET. Then, the validation of real-time PCR showed that the expression of miR-1197 were increased in UOK109 cells upon circMET knockdown (Fig. 7I). Similarly, overexpression of circMET down-regulated the level of miR-1197 in 786-O cells (Fig. 7J).

To verify the biological function of miR-1197 in *NONO-TFE3* tRCC, a series of rescue experiments were performed. Cell proliferation, colony formation and tumor sphere formation were inhibited by transfected with sh-circMET compared with the negative control in UOK109 cells, but the miR-1197 mimics reversed this phenomenon (Fig. 7K-M). Additionally, overexpression of circMET significantly promoted the cell proliferation, colony formation and tumor sphere formation of 786-O cell line. After inhibited the miR-1197, the capacities of cell proliferation, colony formation and tumor sphere formation came back to control level. The data of cell cycle from flow cytometry confirmed it (Fig. 7N). Taken together, these results indicate that circMET functions as a ceRNA to sponge miR-1197.

### CircMET modulates SMAD3 expression through post‑transcriptional regulation

**Fig. 8:**
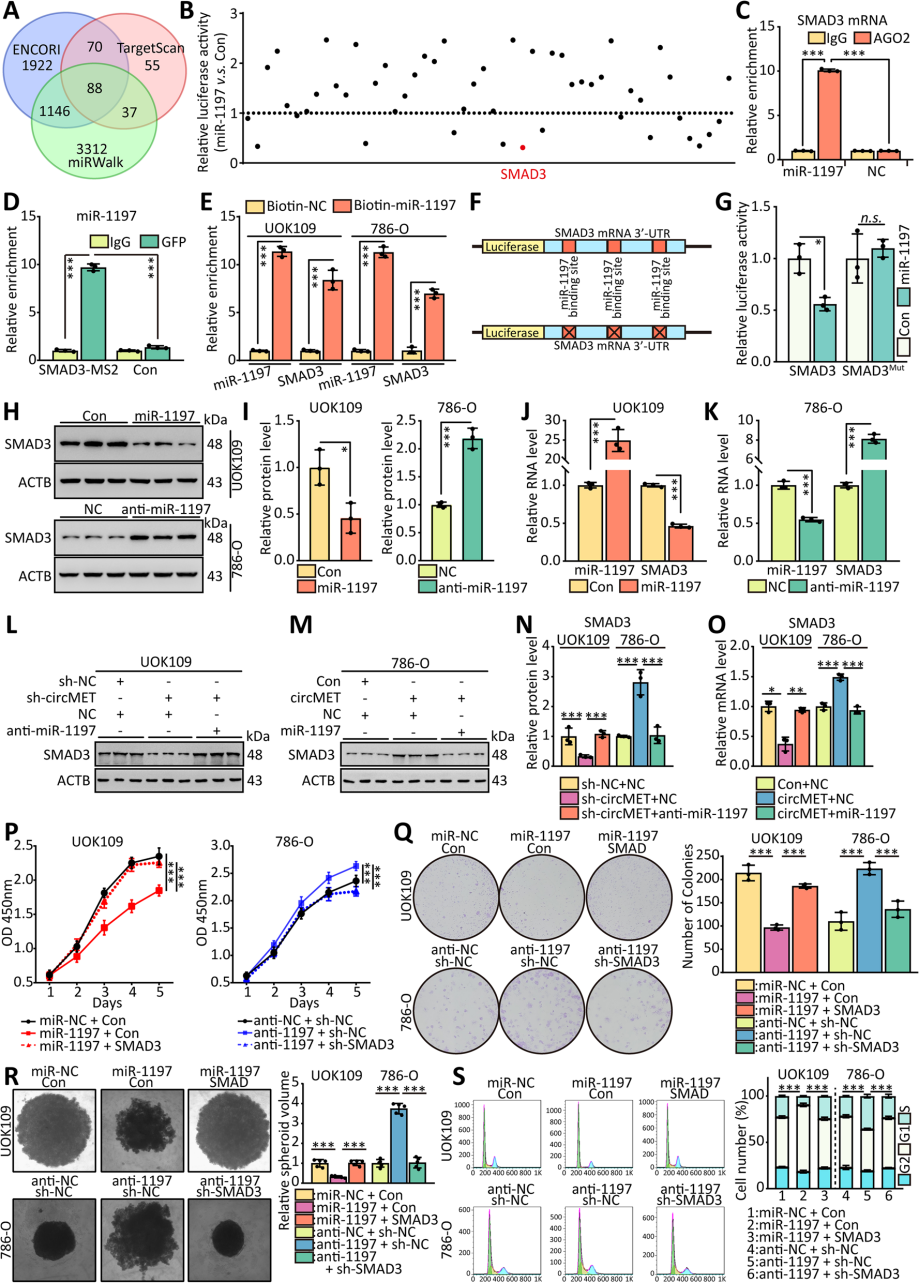
CircMET modulates SMAD3 expression through post‑transcriptional regulation. **(A)** Schematic of the selection for the direct downstream target of miR-1197. **(B)** HEK293T cells were co-transfected with 3’-UTR luciferase truncations of top50 mRNA and miR-1197, and the luciferase activity was determined using a dual luciferase reporter assay after 48 h. **(C)** RIP and MS2-RIP assays were performed using AGO2/GFP antibody in UOK109 cells, then the enrichment of SMAD3 mRNA was detected by real-time PCR. **(E)** The miR-1197-Biotin complex was enriched by Biotin-antibody with protein extracts from UOK109 and 786-O cells. The enrichment of SMAD3 and miR-1197 was detected by real-time PCR. **(F)** Schematic illustration of SMAD3 3’-UTR wild type and mutation luciferase reporter vectors. **(G)** HEK293T cells were co-transfected with miRNA mimics respectively and wild-type or mutant SMAD3 3’-UTR luciferase reporter vector, and luciferase reporter activity was detected. **(H–O)** The RNA levels of miR-1197 and SMAD3 and the protein level of SMAD3 were detected after transfection with indicated lentivirus. **(P)** Cell viability of UOK109 and 786-O cells was determined using CCK-8 assays after transfection for 48 h. **(Q-R)** A colony formation and tumor sphere formation assay were used to determine the colony and tumor sphere formation ability of UOK109 and 786-O cells co-transfected with indicated lentivirus. **(S)** Cell cycle analysis was performed using flow cytometry in cells transfected with indicated lentivirus. The data are presented as the mean ± SD, *n.s.* = non-significant, *P < 0.05, ***P < 0.001

Interestingly, through miRWalk, ENCORI and TargetScan (http://www.targetscan.org/) [40] analysis, 88 genes were found to be potential target genes of miR-1197 (Fig. 8A). To explore which gene could be affected by miR-1197 mostly, the 3’-UTR of top 50 genes was cloned into luciferase reporter plasmid and co-transfected with miR-1197, respectively. The result of dual-luciferase assay indicated the most notably downregulated luciferase activity after overexpression of miR-1197 was SMAD3 (Fig. 8B). We performed RIP assays and found that the ectopic expression of miR-1197 enhanced the enrichment of AGO2 at SMAD3 mRNA (Fig. 8C), and the result of MS2-RIP assay indicated that miR-1197 could bind to exogenous SMAD3 (Fig. 8D). The pulldown assay using biotin-labeled miR-1197 further corroborated this idea (Fig. 8E). In-deed, luciferase reporter assays showed that WT-SMAD3-driven luciferase expression was significantly inhibited by co-transfection with the miR-1197 (Fig. 8F, H). However, this inhibitory effect was abolished by mutation of the putative miR-1197 binding site in the SMAD3 3’-UTR.

Overexpression or downregulation of miR-1197 caused a significant decrease or increase in SMAD3 expression at mRNA and protein levels (Fig. 8H-K). Additionally, we performed rescue assays to evaluate whether circMET regulates SMAD3 by competing for miR-1197. The results showed that suppression of circMET decreased SMAD3 levels and miR-1197 inhibitor impaired this downregulation (Fig. 8L-O), whereas overexpression of circMET increased SMAD3 levels and ectopic expression of miR-1197 repressed this increase. CCK-8, colony formation, tumor sphere formation and flow cytometry assays were performed, and the results revealed that cell proliferation (Fig. 8P-R) and cell cycle (Fig. 8S) were inhibited by overexpression of miR-1197 compared with the negative control in UOK109 cells, and ectopic expression of SMAD3 recovered this increase. The consistent results were observed in 786-O cells. Taken together, these results indicate that miR-1197 can bind to 3’-UTR of SMAD3 mRNA and mediated SMAD3 expression levels in *NONO-TFE3* tRCC.

## Discussion

In a wide range of human malignancies, the patterns of circRNA expression change tremendously during tumourigenesis and cancer progression, and the dysregulated circRNAs are clearly involved in many physiological and pathological processes [9, 11]. A growing number of studies have suggested that circRNA could be an accuracy biomarker of some cancer, but the application of circRNAs in clinic is still need a long time to develop and research under the current situation. In addition to the competing endogenous RNAs (ceRNA) mechanism, much of biological function and underlying molecular mechanism remains to be learned. In this study, we clarified that circMET expression were enhanced by high-expression of NONO-TFE3 fusion protein in *NONO-TFE3* tRCC. Importantly, circMET regulated the expression of CDKN2A, a critical tumor suppressor gene, and SMAD3 which could maintain the stem cell properties of cancer stem-like cells [41–43]. Mechanistically, circMET directly bound to CDKN2A mRNA, thereby promoted the interaction between YTHDF2 and CDKN2A mRNA, which causes the shortened half-life of CDKN2A mRNA. Meanwhile, sponging miR-1197 was the other biological function of circMET. MiR-1197 could directly bind to the 3’-UTR of SMAD3 mRNA and regulated the expression of SMAD3. Last, we demonstrated that circMET promotes tumor progression and silence of circMET can inhibit cell proliferation of tumor. This study explains a novel regulatory mechanism of circMET in *NONO-TFE3* tRCC, which might provide novel insights for better tailoring treatment and identification of new therapeutic targets, such as targeted therapy using small RNA and detection kit based on CRISPR/Cas12. Due to the small sample size of *NONO-TFE3* tRCC in the validation cohort, we were limited to firmly establish the link between circMET upregulation and prognosis. Moreover, we propose to continuously carry out a prospective cohort study to detailly explore the clinical outcome with the expression of circMET, which helps the development of *NONO-TFE3* tRCC diagnosis and treatment. Otherwise, we predicted the possibility of encoding protein of circMET, the possibility of encoding protein is relatively low (Fig. S12A), and overexpression of this potential peptide could not affect the proliferation of 786-O (Fig. S12B, C).

Consistently, the ceRNA network regulation of circRNAs is being referred to so extensively. Recently, several studies proposed a novel mechanism based on circRNA-mRNA interaction. A current study reported that circRNA circNSUN2 interact directly with 3’-UTR of high mobility group AT-hook 2 (HMGA2) mRNA and then inhibit the decay of HMGA2 mRNA, and the stability of HMGA2 mRNA was enhanced via Insulin-Like Growth Factor 2 mRNA-Binding Protein 2 (IGF2BP2) [44]. Besides this, circRNA circCUX1 could promote the decay of caspase 1 mRNA by direct interaction between circRNA-mRNA via AAUAAA motif [45]. Here, we described that circMET interacted with 3’-UTR of CDKN2A mRNA directly via AAUAAA motif and then inhibited CDKN2A expression by promoting the degradation of CDKN2A mRNA. We revealed that the decay of CDKN2A mRNA was in YTHDF2 dependent manner.

Methyltransferase 3 (METTL3)-mediated m^6^A modification is the most widespread base modification in mRNA, which has a broad biological function, while any perturbation in m^6^A levels can result in malfunction or diseases. An increasing number of studies have confirmed that m^6^A writers (METTL3, METTL14, KIAA1429, WTAP), erasers (FTO and ALKBH5) and readers (YTHDC1, YTHDC2, YTHDF1, YTHDF2 and HNRNPC) have distinct functions within different types of cancer cells [46, 47]. M^6^A modifications may play important roles in RNA production, stability, subcellular localization and interactions in cancers [29]. The differential modification sites and m^6^A reader proteins reflect their divergent biological functions. YTHDC1 facilitates mRNA binding to both the canonical export receptor NXF1 and splicing factors SRSF3 mediating export and metabolism of m^6^A-modified mRNAs [29]. On the other hand, it has been reported previously that YTHDC1 could mediate export of m^6^A-modified circRNA [44]. Here, it has been shown that m^6^A labeling promoted the cytoplasmic localization of circMET through recruiting YTHDC1. Surprisingly, the m^6^A modification on circMET could even recruit the YTHDF2 and lead to the decay of CDKN2A mRNA interacted with circMET.

There has been substantial interest in the ceRNA network mechanism in recent years, with many studies in the area revolving around how dysregulation of ceRNA, including long non-coding RNAs (lncRNAs), circRNA, mRNA and miRNA, can influence tumor initiation and progression. In this study, we found that circMET could function as a biological sponge and modulate the expression of SMAD3 through post-transcriptional regulation of miR-1197. Increasing studies revealed the important functions of ceRNA network in tumor proliferation, antiapoptosis, metastasis and other tumor development processes. For example, circ3823 contributes to growth, metastasis and angiogenesis of colorectal cancer by absorbing miR-30c-5p and mediating TCF7 [48]. In addition to this, the resistant tumors showed significant expression changes of circRNAs, CircMEMO1 modulates the promoter methylation by acting as a sponge for miR-106b-5p to regulate sorafenib treatment sensitivity of hepatocellular carcinoma [49]. Although the ceRNA network mechanism is superficial, it proffers an extremely powerful toolkit for clinical diagnostic and therapeutic implications.

## Conclusion

**Fig. 9:**
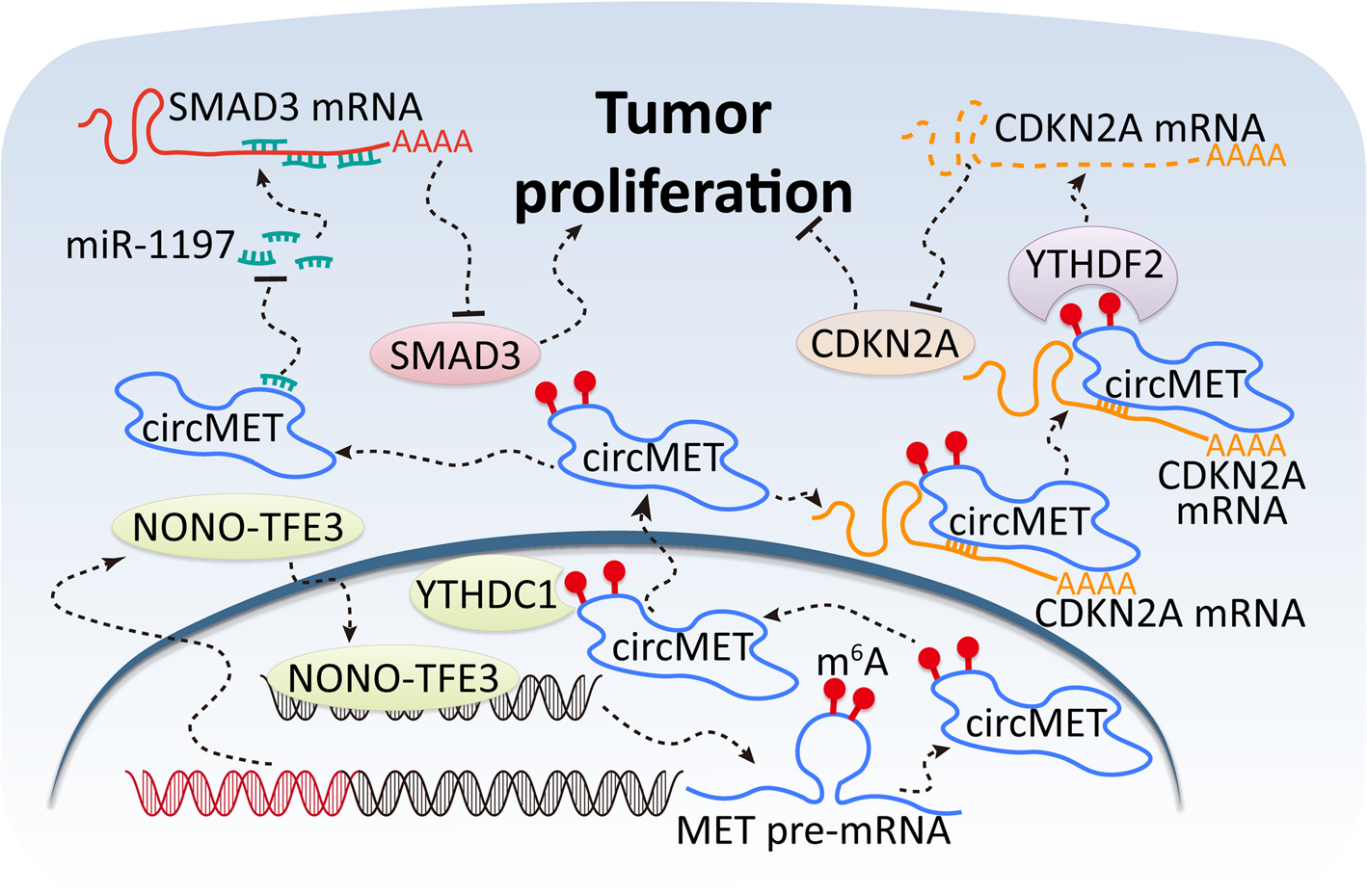
Schematic diagram for the mechanisms of circMET functioning as both an mRNA decay accelerator and a miRNA sponge to promote proliferation of *NONO-TFE3* tRCC

In summary, we observed that the expression of circMET was enhanced by high-expressed NONO-TFE3 fusion protein in *NONO-TFE3* tRCC. YTHDC1 promotes cytoplasmic export of circMET via binding to m^6^A modification. CircMET markedly weakened CDKN2A expression by binding to its mRNA directly to recruit YTHDF2 which led to mRNA decay of CDKN2A, and enhanced the expression of SMAD3 through absorbing miR-1197 (Fig. 9). Our findings help better understand the biological function of circMET in *NONO-TFE3* tRCC pathogenesis and provide a rationale for RNA-based diagnosis and treatment in Xp11.2 tRCC and other human malignancies.

## Supplementary Information

**Additional file 1:** **Figure S1. **The RNA level of MET mRNA and circRNAs derived from MET gene in RCC and the structural features of circMET. **Figure S2.** The RNA level of circMET and MET mRNA in UOK109 (A) and 786-O (B) after transfected with indicated lentivirus. **Figure S3.** ChIP assay was performed after UOK109 cells transfected indicated lentivirus. **Figure S4.** The transcription of MET gene is enhanced by NONO-TFE3. **Figure S5.** The m6A methylation is involved in the export of circMET from the nucleus. **Figure S6.** The function of m6A modification on exon 2 of MET mRNA. **Figure S7.** The m6A modification on circMET promotes tumor proliferation. **Figure S8.** The stability of GAPDH mRNA in cells transfected with indicated lentivirus after treatment with α-amanitin. **Figure S9.** CircMET mediates NONO-TFE3 tRCC proliferation through CDKN2A. **Figure S10.** CircMET mediates the RNA level of CDKN2A through YTHDF2. **Figure S11.** CircMET recruits YTHDF2 to CDKN2A mRNA via m6A methylation. **Figure S12.** The function of potential peptide encoded by circMET. **Figure S13.** Relationship between circMET and CDKN2A and SMAD3 mRNA in NONO-TFE3 tRCC (A) and ccRCC (B). **Figure S14.** The function of m6A modification on exon 2 of MET mRNA. **Figure S15.** Relationship between circMET and AKT1, CCNB1 and CCND1 mRNA. **Table S1.** Primers used for real-time PCR. **Table S2.** Primers used for ChIP assay and MeRIP. **Table S3**. Probes used for RNA FISH. **Table S4.** ShRNA used for silencing target genes. **Table S5.** Guide RNA used for dCas9-ChIP system and targeted RNA methylation system. **Table S6.** Primary antibodies used in this study.

## Data Availability

The datasets used and/or analyzed during the current study are available from the corresponding author on reasonable request.

## Abbreviations

ACTB: Actin, beta
ALKBH5: AlkB homolog 5, RNA demethylase
AGO2: Argonaute 2
CCK8: Cell counting kit-8
ChIP: Chromatin immunoprecipitation
CRISPR: Clustered regularly interspaced short palindromic repeats
FISH: Fluorescence in situ hybridization
FTO: Fat mass and obesity associated
GFP: Green fluorescent protein; m^6^A: *N*^6^-methyladenosine
METTL3: Methyltransferase like 3
MiT/TFE: Microphthalmia family of bHLH-LZ transcription factors
NES: Nuclear export signal
NLS: Nuclear localization signal
NONO: Non-POU domain containing octamer binding
PI: Propidium iodide
RIP: RNA immunoprecipitation
TFE3: Transcription factor binding to IGHM enhancer 3
TRAF3IP2-AS1: TRAF3IP2 antisense RNA 1
tRCC: Translocation renal cell carcinoma
YTHDC: YTH domain-containing protein 1
YTHDF: YTH N6-methyladenosine RNA binding protein
