# Supplementary material for: CircMET promotes tumor proliferation by enhancing CDKN2A mRNA decay and upregulating SMAD3
Source: Mol Cancer. 2022 Jan 18;21:23. doi: 10.1186/s12943-022-01497-w (PMC8764797; doi:10.1186/s12943-022-01497-w)
Supplement: Supplementary file 1 — Additional file 1: Figure S1. The RNA level of MET mRNA and circRNAs derived from MET gene in RCC and the structural features of circMET. Figure S2. The RNA level of circMET and MET mRNA in UOK109 (A) and 786-O (B) after transfected with indicated lentivirus. Figure S3. ChIP assay was performed after UOK109 cells transfected indicated lentivirus. Figure S4. The transcription of MET gene is enhanced by NONO-TFE3. Figure S5. The m6A methylation is involved in the export of circMET from the nucleus. Figure S6. The function of m6A modification on exon 2 of MET mRNA. Figure S7. The m6A modification on circMET promotes tumor proliferation. Figure S8. The stability of GAPDH mRNA in cells transfected with indicated lentivirus after treatment with α-amanitin. Figure S9. CircMET mediates NONO-TFE3 tRCC proliferation through CDKN2A. Figure S10. CircMET mediates the RNA level of CDKN2A through YTHDF2. Figure S11. CircMET recruits YTHDF2 to CDKN2A mRNA via m6A methylation. Figure S12. The function of potential peptide encoded by circMET. Figure S13. Relationship between circMET and CDKN2A and SMAD3 mRNA in NONO-TFE3 tRCC (A) and ccRCC (B). Figure S14. The function of m6A modification on exon 2 of MET mRNA. Figure S15. Relationship between circMET and AKT1, CCNB1 and CCND1 mRNA. Table S1. Primers used for real-time PCR. Table S2. Primers used for ChIP assay and MeRIP. Table S3. Probes used for RNA FISH. Table S4. ShRNA used for silencing target genes. Table S5. Guide RNA used for dCas9-ChIP system and targeted RNA methylation system. Table S6. Primary antibodies used in this study. [file 12943_2022_1497_MOESM1_ESM.pdf]

CircMET promotes tumor proliferation by enhancing CDKN2A mRNA decoy and upregulating SMAD3

Lei Yang[1,2#], Yi Chen[1,2#], Ning Liu[1,2], Yanwen Lu[1,2], Zhenhao Yang[3], Weidong Gan[4*], Dongmei Li[1,2*]

1. Immunology and Reproduction Biology Laboratory & State Key Laboratory of Analytical Chemistry for Life Science, Medical School, Nanjing University, Nanjing, Jiangsu 210093, China

2. Jiangsu Key Laboratory of Molecular Medicine, Nanjing University, Nanjing, Jiangsu 210093, China

3. Department of Urology, Nanjing Drum Tower Hospital Clinical College of Traditional Chinese and Western Medicine, Nanjing University of Chinese Medicine, Nanjing, Jiangsu 210008, China.

4. Department of Urology, Nanjing Drum Tower Hospital, The Affiliated Hospital of Nanjing University Medical School, Nanjing, Jiangsu 210008, China.

[#]These authors contributed equally to this work.

* Corresponding Author:

Dongmei Li, Immunology and Reproduction Biology Laboratory & State Key Laboratory of Analytical Chemistry for Life Science, Medical School, Nanjing University, Nanjing, Jiangsu 210093, China.

Email: lidm@nju.edu.cn

Weidong Gan, Department of Urology, Affiliated Drum Tower Hospital of Medical School of Nanjing University, Nanjing, Jiangsu 210008, China.

Email: gwd@nju.edu.cn

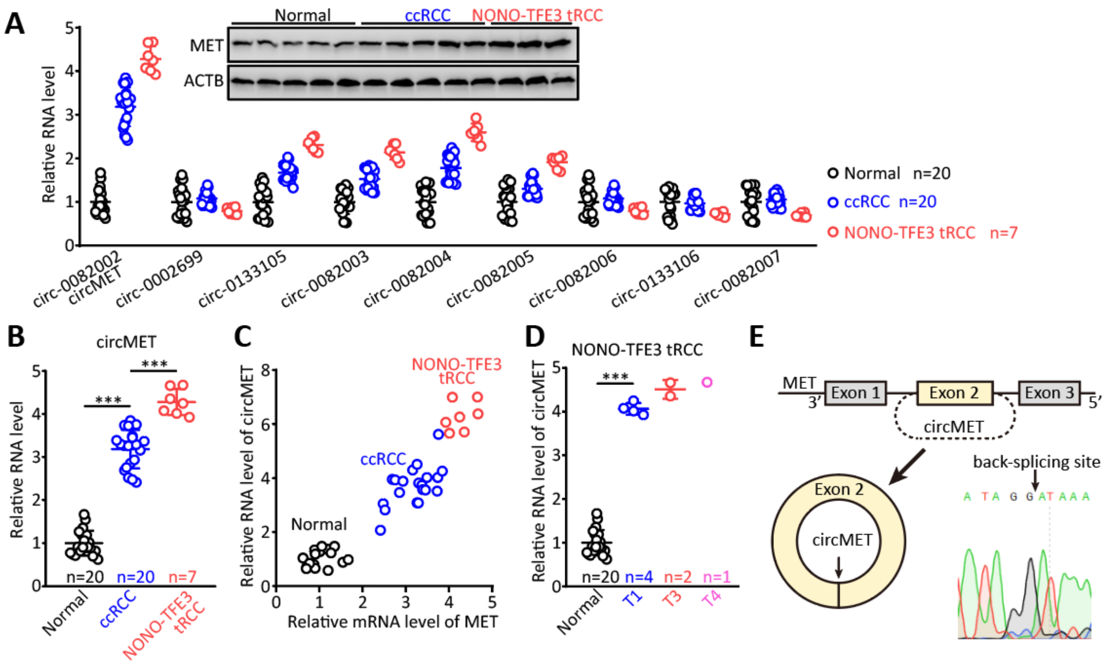

Figure S1. The RNA level of MET mRNA and circRNAs derived from MET gene in RCC and the structural features of circMET. (A) The RNA level of circRNAs derived from MET gene in nontumor tissues, ccRCC and NONO-TFE3 tRCC. **(B)** The RNA level of circMET in nontumor tissues, ccRCC and NONO-TFE3 tRCC. **(C)** A positive correlation between circMET and MET was observed in RCC tissues. **(D)** The correlation between circMET expression and the clinical stage were analyzed. **(E)** Scheme illustrating the production of circMET. The data are presented as the mean ± SD, ***$P< 0.001$

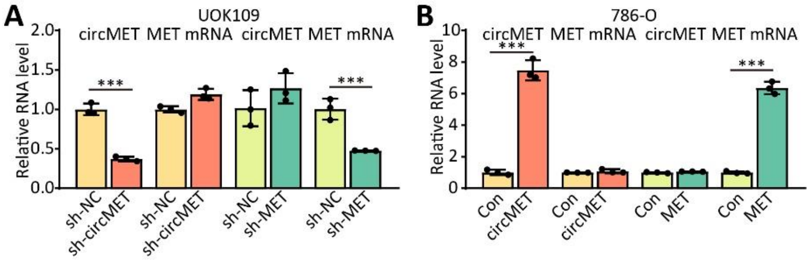

Figure S2. The RNA level of circMET and MET mRNA in UOK109 **(A)** and 786-O **(B)** after transfected with indicated lentivirus. The data are presented as the mean ± SD, ***P< 0.001

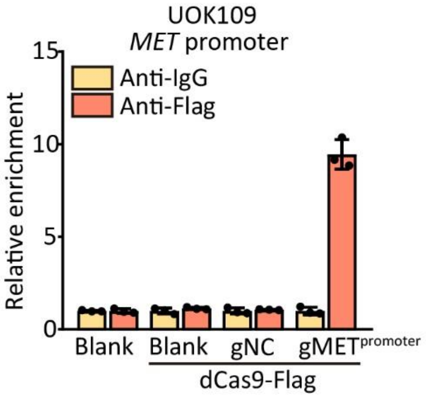

Figure S3. ChIP assay was performed after UOK109 cells transfected indicated lentivirus.

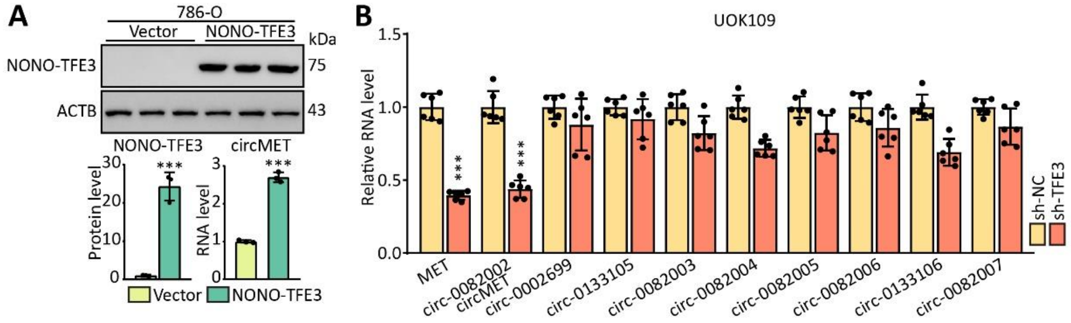

Figure S4. The transcription of MET gene is enhanced by NONO-TFE3. **(A)** The protein level of NONO-TFE3 and the circMET expression levels were detected after transfection with NONO-TFE3 plasmids for 48h. **(B)** The RNA level of circRNAs derived from MET gene was detected after transfected with sh-TFE3. The data are presented as the mean ± SD, ****P*< 0.001

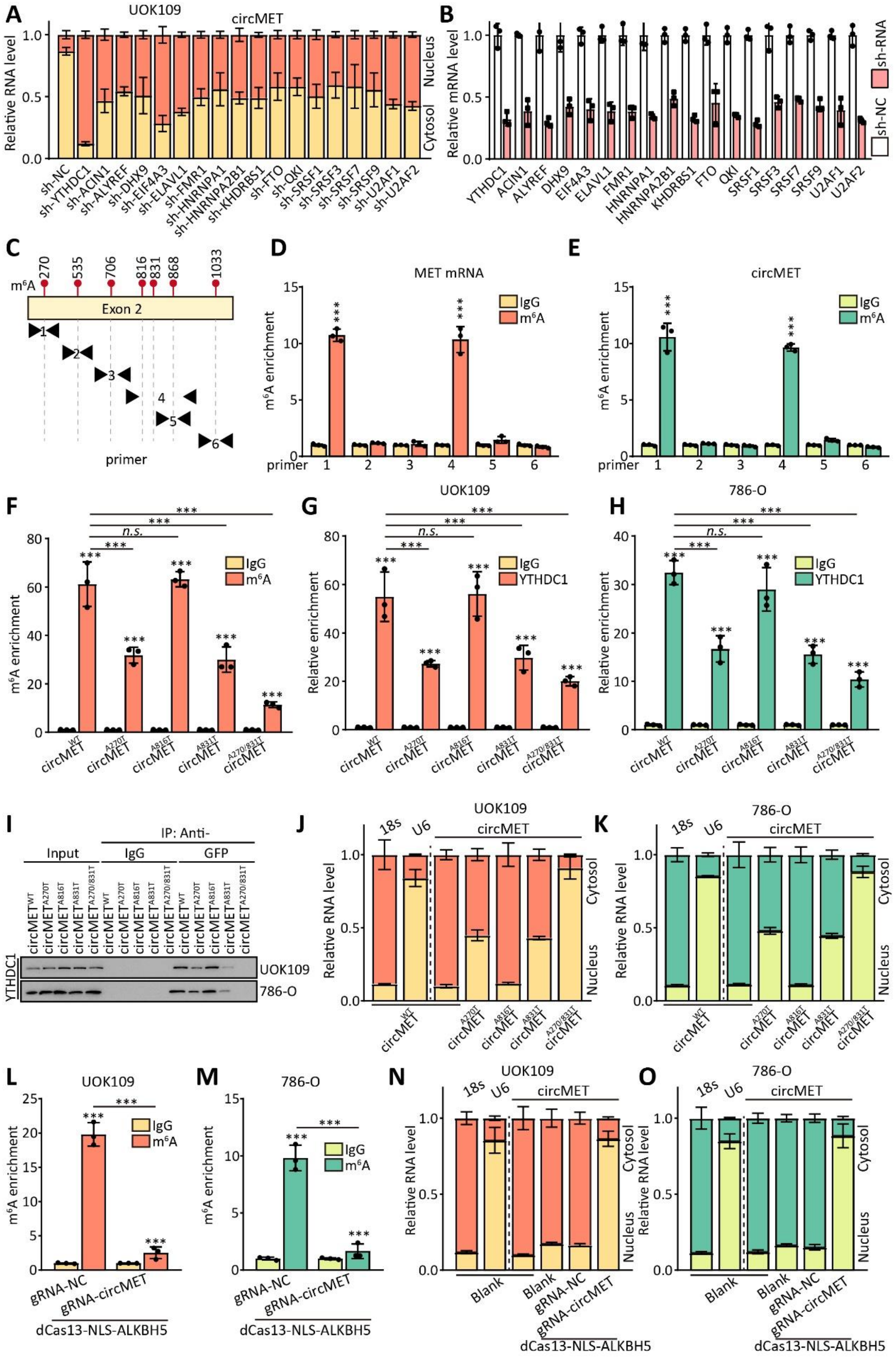

Figure S5. The m^{6}A methylation is involved in the export of circMET from the nucleus. **(A)** The subcellular distribution of circMET was analyzed via real-time PCR

in UOK109 cells after transfected with indicated lentivirus. **(B)** The RNA level was detected via real-time PCR in UOK109 cells after transfected with indicated lentivirus. **(C)** Schematic diagram of the potential m^6A methylation locus. **(D)** Abundance of MET fragment among RNA immunoprecipitated with anti-m^6A antibody in UOK109 cells was measured by real-time PCR and normalized to IgG. **(E)**The total RNA isolated from UOK109 cells was treated with RNase R, and the MeRIP was performed. Abundance of circMET fragment among RNA immunoprecipitated with anti-m^6A antibody was measured by real-time PCR and normalized to IgG. **(F)** The 786-O cells were transfected with circMET contained indicated mutation, and the abundance of circMET among RNA immunoprecipitated with anti-m^6A antibody was measured by real-time PCR and normalized to IgG. **(G-H)** The UOK109 **(G)** and 786-O **(H)** cells were transfected with circMET contained indicated mutation, and the RIP assay was performed with YTHDC1 antibody. Abundance of circMET was measured by real-time PCR. **(I)** The UOK109 cells were transfected with MS2-GFP plasmid, circMET contained 6×MS2 stem loop and indicated mutation, and the MS2-RIP assay was performed with GFP antibody. Abundance of YTHDC1 was measured by western blot. **(J-K)** The subcellular distribution of circMET was analyzed via real-time PCR in UOK109 and 786-O cells after transfected with indicated lentivirus. U6 and 18s rRNA were used as nuclear and cytoplasmic markers, respectively. **(L-M)** The UOK109 **(L)** and 786-O **(M)** cells were transfected with indicated lentivirus, and the MeRIP assay was performed. Abundance of circMET was measured by real-time PCR. **(N-O)** The subcellular distribution of circMET was analyzed via real-time PCR in UOK109 and 786-O cells after transfected with indicated lentivirus. U6 and 18s rRNA were used as nuclear and cytoplasmic markers, respectively. The data are presented as the mean ± SD, $^{***}P<0.001$

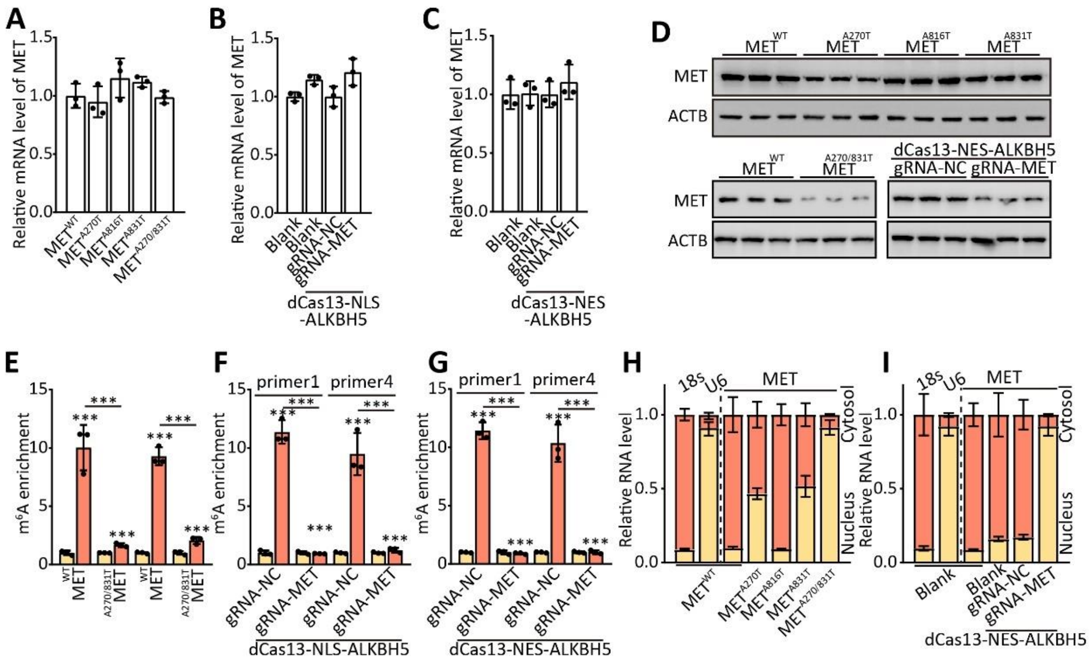

Figure S6. The function of m^{6}A modification on exon 2 of MET mRNA. **(A-C)** The UOK109 cells were transfected with indicated lentivirus, and the RNA level of MET mRNA was measured by real-time PCR. **(D)** The UOK109 cells were transfected with indicated lentivirus, and the protein level of MET was measured by western blot. **(E-F)** The UOK109 cells were transfected with indicated lentivirus, and the abundance of circMET among RNA immunoprecipitated with anti-m^{6}A antibody was measured by real-time PCR and normalized to IgG. **(H-I)** The subcellular distribution of circMET was analyzed via real-time PCR in UOK109 cells after transfected with indicated lentivirus. U6 and 18s rRNA were used as nuclear and cytoplasmic markers, respectively. The data are presented as the mean ± SD, ***$P < 0.001$

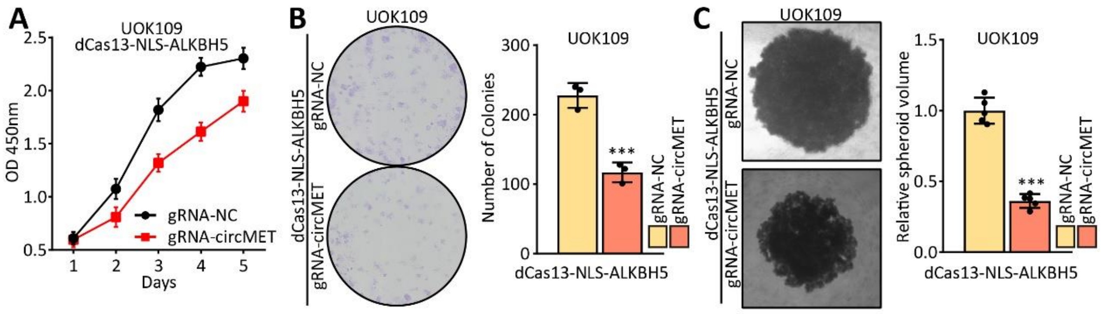

Figure S7. The m^{6}A modification on circMET promotes tumor proliferation. **(A)** Cell viability of UOK109 and 786-O cells was determined using CCK-8 assays after transfection for 48h. **(B-D)** A colony formation and tumor sphere formation assay were used to determine the colony and tumor sphere formation ability of UOK109 and 786-O cells co-transfected with indicated lentivirus. The data are presented as the mean ± SD, ****P*< 0.001

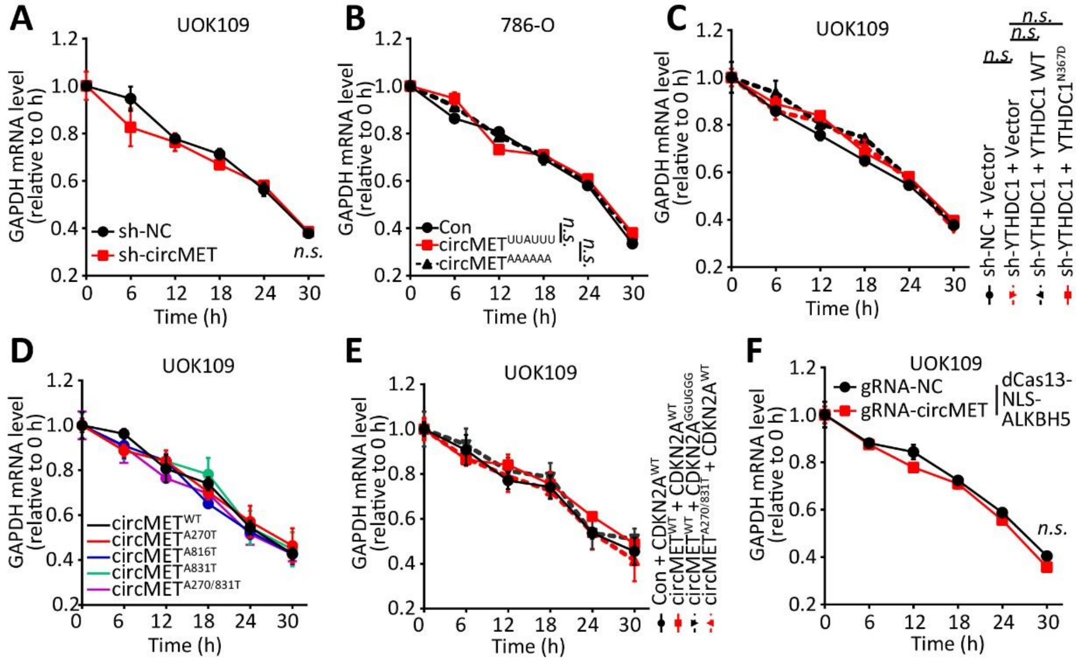

Figure S8. The stability of GAPDH mRNA in cells transfected with indicated lentivirus after treatment with α-amanitin. The data are presented as the mean ± SD

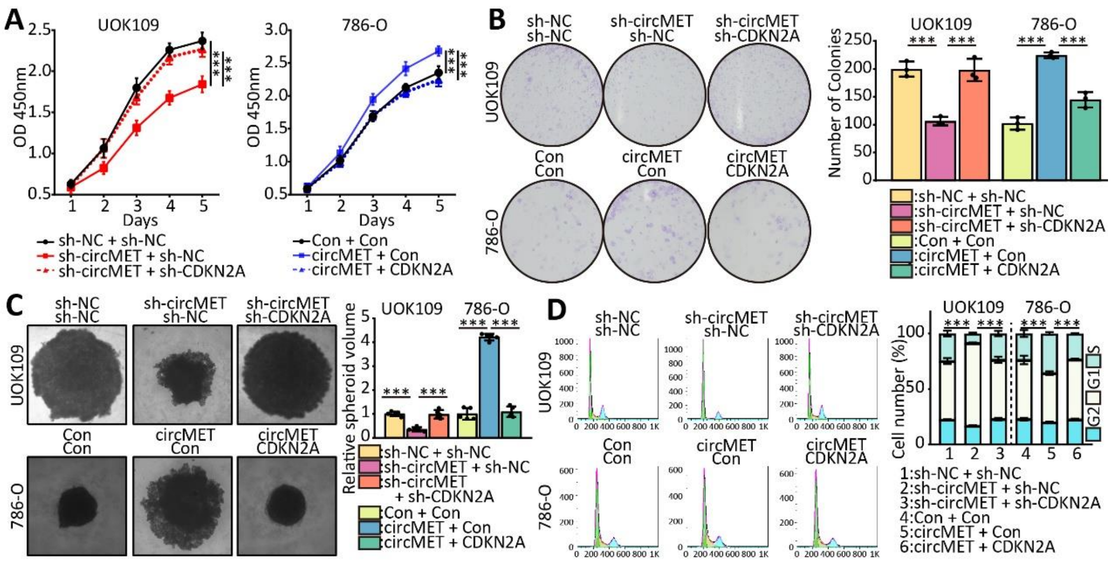

Figure S9. CircMET mediates *NONO-TFE3* tRCC proliferation through CDKN2A. **(A)** Cell viability of UOK109 and 786-O cells was determined using CCK-8 assays after transfection for 48h. **(B-C)** A colony formation and tumor sphere formation assay were used to determine the colony and tumor sphere formation ability of UOK109 and 786-O cells co-transfected with indicated lentivirus. **(D)** Cell cycle analysis was performed using flow cytometry in cells transfected with indicated lentivirus. The data are presented as the mean ± SD, $^{**}P < 0.01$, $^{***}P < 0.001$

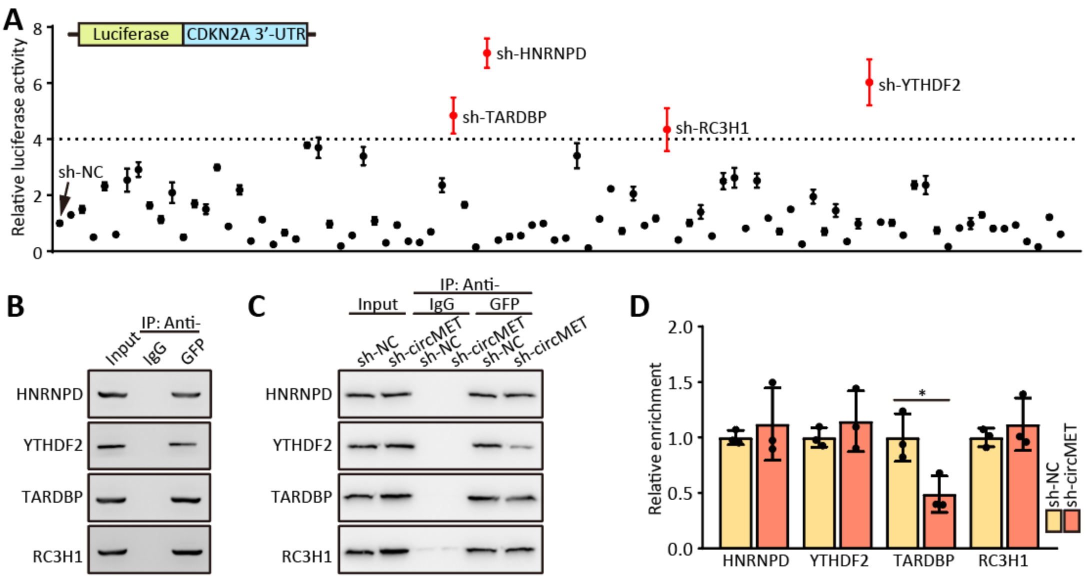

Figure S10. CircMET mediates the RNA level of CDKN2A through YTHDF2. **(A)** HEK293T cells were co-transfected with indicated mRNA 3'-UTR luciferase truncations and shRNAs, and the luciferase activity was determined using a dual luciferase reporter assay after 48h. **(B)** MS2-RIP assays were performed to confirm the association of indicated protein with circMET. **(C-D)** The UOK109 cells were transfected with MS2-GFP plasmid, CDKN2A contained 6×MS2 stem loop and indicated shRNAs, and the MS2-RIP assay was performed with GFP antibody. Abundance of YTHDF2 was measured by western blot. The data are presented as the mean ± SD, **P*< 0.05

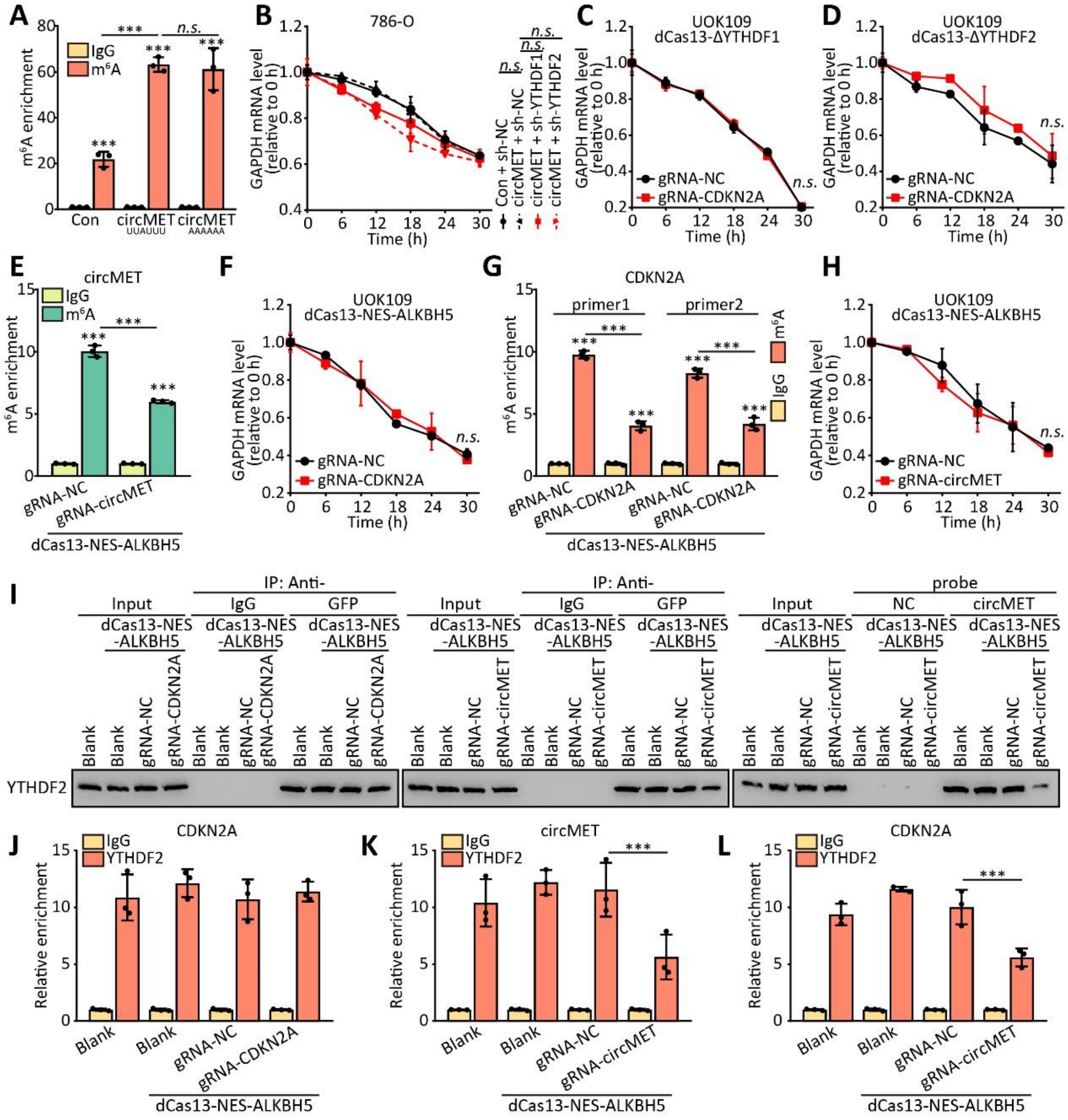

Figure S11. CircMET recruits YTHDF2 to CDKN2A mRNA via m^6A methylation. **(A)** The UOK109 cells were transfected with indicated lentivirus, and the abundance of circMET among RNA immunoprecipitated with anti-m^6A antibody was measured by real-time PCR and normalized to IgG. **(B-D)** The stability of GAPDH mRNA in cells transfected with indicated lentivirus after treatment with α-amanitin. **(E)** The UOK109 cells were transfected with indicated lentivirus, and the abundance of circMET among RNA immunoprecipitated with anti-m^6A antibody was measured by real-time PCR and normalized to IgG. **(G)** The stability of GAPDH mRNA in cells transfected with indicated lentivirus after treatment with α-amanitin. **(F)** The UOK109 cells were transfected with indicated lentivirus, and the abundance of CDKN2A

among RNA immunoprecipitated with anti-m^{6}A antibody was measured by real-time PCR and normalized to IgG. **(H)** The stability of GAPDH mRNA in cells transfected with indicated lentivirus after treatment with α-amanitin. **(I)** The UOK109 cells were transfected with indicated lentivirus, and the MS2-RIP assay and RNA pulldown were performed with GFP antibody or circMET probe. Abundance of YTHDF2 was measured by western blot. **(J-K)** The UOK109 cells were transfected with indicated lentivirus, and the RIP assay was performed with YTHDF2 antibody. Abundance of circMET or CDKN2A was measured by real-time PCR.The data are presented as the mean ± SD, ***P< 0.001

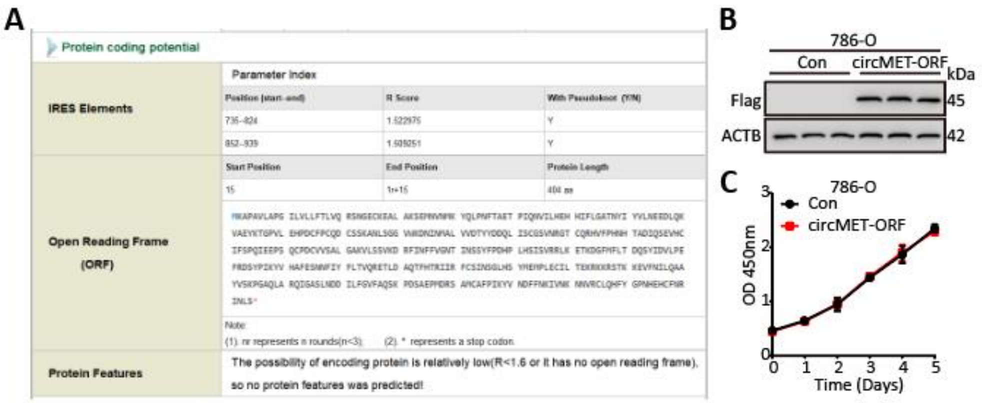

Figure S12. The function of potential peptide encoded by circMET. **(A)** The possibility of encoding protein of circMET was predicted. **(B)** The potential peptide encoded by circMET was overexpressed in 786-O cells. **(C)** Cell viability of UOK109 cells was determined using CCK-8 assay after transfection for 48h. The data are presented as the mean ± SD

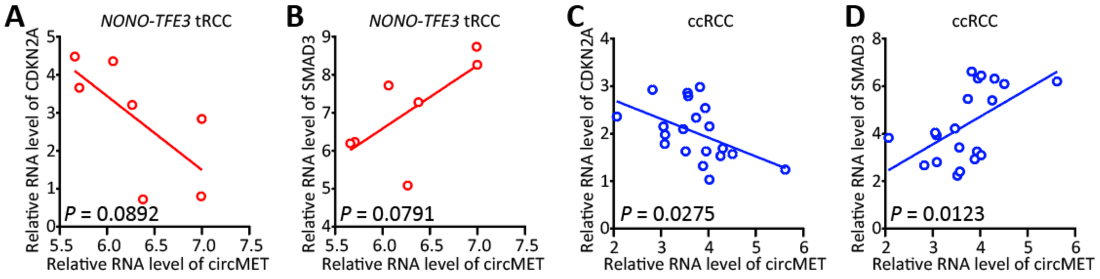

Figure S13. Relationship between circMET and CDKN2A and SMAD3 mRNA in *NONO-TFE3* tRCC **(A)** and ccRCC **(B)**.

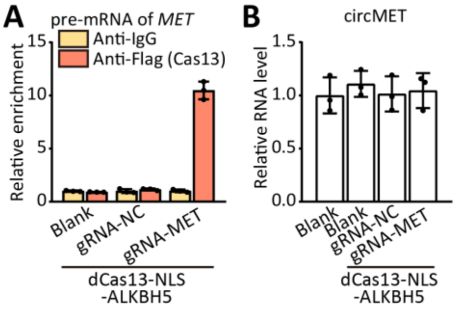

Figure S14. The function of m^{6}A modification on exon 2 of MET mRNA. **(A)** The UOK109 cells were transfected with indicated lentivirus, and the abundance of pri-mRNA of *MET* among RNA immunoprecipitated with anti-Flag antibody was measured by real-time PCR and normalized to IgG. **(B)** The UOK109 cells were transfected with indicated lentivirus, and the RNA level of circMET was measured by real-time PCR.

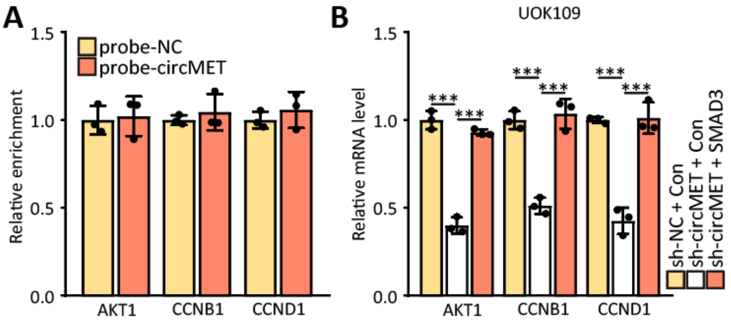

Figure S15. Relationship between circMET and AKT1, CCNB1 and CCND1 mRNA. **(A)** RNA pulldown assay was performed to confirm the association of AKT1, CCNB1 and CCND1 mRNA with circMET. **(B)** The RNA level of AKT1, CCNB1 and CCND1 mRNA in UOK109 cells after transfected with indicated lentivirus. The data are presented as the mean ± SD, ****P*< 0.001

Table S1. Primers used for real-time PCR.

Target gene	Primer sequence (5'-3') Forward	Reverse	Size (bp)
circMET	AGCACTGCTTTAATAGGATAAACC	TCGGACTTTGCTAGTGCCTC	123
CDKN2A	CTTCCTCGGGTGCCGATAC	ACCCCTTCATTGCTACTCGAT	153
SMAD3	GCGTGCGGCTCTACTACATC	GCACATTCGGGTCAACTGGTA	233
18s rRNA	CAGCCACCCGAGATTGAGCA	TAGTAGCGACGGGCGGTGTG	252
GAPDH	AACGGATTTGGTCGTATTGGG	CCTGGAAGATGGTGATGGGAT	211
AKT1	TCCTCCTCAAGAATGATGGCA	GTGCGTTCGATGACAGTGGT	181
CCNB1	AATAAGGCGAAGATCAACATGGC	TTTGTTACCAATGTCCCCAAGAG	111
CCND1	CAATGACCCCGCACGATTTC	CATGGAGGGCGGATTGGAA	146
circMET ▷◁	GGTTCACTGCATATTCTCCCC	ACCATCTTTCGTTTCCTTTAGCC	205
circMET ◁▷	AGCACTGCTTTAATAGGATAAACC	TCGGACTTTGCTAGTGCCTC	123
GAPDH ▷◁	AACGGATTTGGTCGTATTGGG	CCTGGAAGATGGTGATGGGAT	211
GAPDH ◁▷	CCCAATACGACCAAATCCGTT	ATCCCATCACCATCTTCCAGG	NA
TFE3	TGCCTGTGTCAGGGAATCTG	CGACGCTCAATTAGGTTGTGAT	184

Table S2. Primers used for ChIP assay and MeRIP.

Target promoter	Primer sequence (5'-3') Forward	Reverse	Size (bp)
MET site1	GCTGAGGCCTCTTGGTATGG	AGTGATTTCAGTCCTCAAAAGAGA	229
MET site2	AGAGGTAATCTCTTTTGAGGACTGA	CGTTCCGGCTCTCATACCAA	248
MET site3	AGTTTCACCTTGTCGTGGGC	CCAGGCGACCAGACTGAG	224

Target RNA	Primer sequence (5'-3') Forward	Reverse	Size (bp)
MET primer 1	CAGAGGAGCAATGGGGAGTG	ATCTGGGTGTTCCAGCACAG	225
MET primer 2	CAGTCGGAGGTTCACTGCAT	AAAGGACTTTGGCTCCCAGG	94
MET primer 3	TGGGAGCCAAAGTCCTTTCA	TGCTTTCAAAGGCATGGACA	228
MET primer 4	TCCATGCCTTTGAAAGCAACA	ACAGAACCTGATTATTCTTGTGTGA	98
MET primer 5	TCACACAAGAATAATCAGGTTCTGT	CATCATTCAGGCTGGCTCCT	197
MET primer 6	AGGAGCCAGCCTGAATGATG	AGCAGTGCTCATGATTGGGT	194
CDKN2A Primer 1	CCAGGTCATGATGATGGGCA	TGCAGCACCACCAGCG	147
CDKN2A Primer 2	ACACGCTGGTGGTGCTG	AATCGGGGATGTCTGAGGGA	195

Table S3. Probes used for RNA FISH.

Target transcript	Probe sequence (5'-3')
circMET probe1	TGAGAGGTTTATCCTATTAAAGCAG
circMET probe2	GAGAGGTTTATCCTATTAAAGCAGTGCT
U6 probe	TTTGCGTGTCATCTTCG
18s rRNA probe	CTGCCTTCCTTGGATGTGGGTAGCCGTTTC

Table S4. ShRNA used for silencing target genes.

Target transcript	Sequence (5'-3')
circMET	GCTTTAATAGGATAAACCTCT
YTHDF1	CCCTACCTGTCCAGCTATTAC
YTHDF2	GATGGATTAAACGATGATGAT
YTHDC1	TGCCTCCAGAGAACCTTATAA
SMAD3	GAGCCTGGTCAAGAAACTCAA
CDKN2A	AGTAACCATGCCCGCATAGAT
TFE3	CAGCTCCGAATTCAGGAACTA
ACIN1	AGCAAGATGAGCTGGATTATC
ADAR	GCCCACTGTTATCTTCACTTT
ALYREF	CGTGGAGACAGGTGGGAAACT
AUH	GCCCACTGTTATCTTCACTTT
BCCIP	GCCTTCTCCTAAGTGAAAGAT
BUD13	CGAGTATCTGAAGCGTTACTT
CELF2	CGCAGAGTAAAGGTTGTTGTT
CNBP	ATTGAGGCTACAGCCTAATTA
CSTF2T	CAAAGGCAGAGTATCCTGATT
DDX54	CCCGGTGTTCAAAGGCATCAT
DGCR8	CCAATCAGAAGCTCATTACTT
DHX9	GAAGGATTACTACTCAAGAAA
DICER1	GCCCACTGTTATCTTCACTTT
DKC1	CACTATACACCTCTTGCATGT
EIF4A3	GCCCACTGTTATCTTCACTTT
ELAVL1	GCAGCATTGGTGAAGTTGAAT
ELAVL3	CCGTGATTTCACCACCAACAA
EWSR1	TGCATTGACTACCAGATTTAT
FAM120A	GCCTTGAATAATGACTCTAAA
FBL	CCTTGAGCCATATGAAAGAGA
FKBP4	GCATGGAGAAAGGAGAACATT
FMR1	GCCCACTGTTATCTTCACTTT
FTO	TCACCAAGGAGACTGCTATTT
FUS	ATGAATGCAACCAGTGTAAGG
FXR1	CGCCAGGTTCCATTTAATGAA
FXR2	GCGGATGATGAAGGGAGATTT
GTF2F1	GCGCAAGATGATCAACGACAA
HNRNPA1	AGATATTTGTTGGTGGCATTA
HNRNPA2B1	CAGAAATACCATACCATCAAT
HNRNPC	GCGCTTGTCTAAGATCAAATT
HNRNPD	AGAGTGGTTATGGGAAGGTAT
HNRNPK	TGATGTTTGATGACCGTCGCG
HNRNPL	GCCGACAACCAAATATACATT
HNRNPM	CTGTGCAAGCTATATCTATGT
HNRNPU	CAGTGCTTCTTCCCTTACAAT
HNRNPUL1	GCCCGCAAGAAACGCAACTAT
IGF2BP1	GCAGTGGTGAATGTCACCTAT
IGF2BP2	AGTGAAGCTGGAAGCGCATAT
IGF2BP3	CGGTGAATGAACTTCAGAATT
KHDRBS1	GTACCGGATATGATGGATGAT
KHDRBS2	GCTCTCAGAAAGAGTACTGAT
KHDRBS3	GCTGGGACAGAAAGTGTTAAT
LARP4B	CAGGCTATCTAGCTTGATAAT
LARP7	CTTGAGCTGTTCTTGGGAGAT
LIN28	TGCTACAACTGTGGAGGTCTA
LIN28A	GCACCAGAGTAAGCTGCACAT
LIN28B	AGAGCATGCAGAAGCGCAGAT
MBNL2	CGGCTATTAGCTTTGCTCCTT
MOV10	CGTTACTGCATCACCAAACTT
MSI1	CACGTTTGAGAGTGAGGACAT
NOP56	CCTGGCAAACAAATGCAGTAT
NOP58	TTAGTTGGAGCACGGCTTATT
NUMA1	CCTTGAAGAGAAGAACGAAAT
PCBP2	CCTGGCTCAATATCTAATCAA
PRPF8	CGCCTCATGAAACATGATGTT
PTBP1	CTCAACGTCAAGTACAACAAT
PUM2	CCTAATCCTACAGCTAATAAA

QKI	CTGATGCTGTGGGACCTATTG
RANGAP1	CTGCCTTCCTAAAGGTGTCAT
RBFOX2	CGGGTTCGTAACTTTCGAGAA
RBM10	CTTCGCCTTCGTCGAGTTTAG
RBM47	GATGAAGAAGCGCGAGGAAAT
RBM5	CCCAGACCTAAGTTTGAAGAT
RBM6	CAAATGTAGAGGAGCATTCTT
RC3H1	CGTGTTGTAAACTCTCAGTAT
RNF219	CCAGTTCTTGTCCAGTAACTA
RTCB	AGAGGCTCCTGAGTCCTATAA
SF3A3	CGAGACACTGAAAGGAACAAA
SF3B4	CCCTGAGATTGATGAGAAGTT
SLTM	CTAGATACAGATGCACGATTT
SMNDC1	CTGGTAAAGTTGGAGTAGGAA
SND1	GCCAAAGGAAACTTGCCTTAT
SRSF1	GAAGCAGGTGATGTATGTTAT
SRSF10	CGTGATGCTGAAGACGCTTTA
SRSF3	TGGAACTGTCGAATGGTGAAA
SRSF7	GAACTGTATGGATTGCGAGAA
SRSF9	TGGAAGAAATGGTTATGATTA
TAF15	GCTCGAAGGAATTCCTGCAAT
TARDBP	GCTCTAATTCTGGTGCAGCAA
TIA1	GCTCTAATTCTGCAACTCTTT
TIAL1	CTTCAGTTGTTCAGTCAGATT
TRA2A	CGTCGAGATTCTTACTATGAT
U2AF1	GAAAGTGTTGTAGTTGATTGA
U2AF2	CGACGAGGAGTATGAGGAGAT
UPF1	GCCTACCAGTACCAGAACATA
ZC3H7B	CGAGATCTGCTTTGACAGTAA

Table S5. Guide RNA used for dCas9-ChIP system and targeted RNA methylation system.

Target promoter	gRNA sequence (5'-3')
$MET^{promoter}$ gRNA1	TGGCAGGGCAGCGCGCGTGT
$MET^{promoter}$ gRNA2	CCAGTGACTCAGCCGGGCAT
$MET^{promoter}$ gRNA3	GCCCGGCTGAGTCACTGGCA
**Target transcript**	**gRNA sequence (5'-3')**
circMET gRNA1	GCTTTAATAGGATAAACCTCTCA
circMET gRNA2	GCACTGCTTTAATAGGATAAACC
circMET gRNA3	CTGCTTTAATAGGATAAACCTCT
CDKN2A gRNA1	ATCTACGTTAAAAGGCAGGACAT
CDKN2A gRNA2	GACATCGCGATGGCCCAGCTCCT
CDKN2A gRNA3	ACATCGCGATGGCCCAGCTCCTC

Table S6. Primary antibodies used in this study.

Source	Primary antibodies	Catalog no.	Working dilution
Sigma-Aldrich	Anti-TFE3 antibody produced in rabbit	HPA023881	ChIP: 5μg
ProteinTech	Anti-TFE3 antibody produced in rabbit	14480-1-AP	WB: 1:2000
ProteinTech	Anti-SMAD3 antibody produced in rabbit	25494-1-AP	WB: 1:1000
Abcam	Anti-CDKN2A antibody produced in mouse	ab109520	WB: 1:1000
ProteinTech	Anti-YTHDC1 antibody produced in rabbit	14392-1-AP	WB: 1:1000 RIP: 5μg
ProteinTech	Anti-YTHDF1 antibody produced in rabbit	17479-1-AP	WB: 1:1000
ProteinTech	Anti-YTHDF2 antibody produced in rabbit	24744-1-AP	WB: 1:2500 RIP: 5μg
ProteinTech	Anti-Flag antibody produced in rabbit	80010-1-RR	WB: 1:2500 ChIP: 5μg
ProteinTech	Anti-GFP antibody produced in rabbit	50430-2-AP	WB: 1:2500 RIP: 5μg
ABclonal	Anti-ACTB antibody produced in rabbit	AC026	WB: 1:15000
Abcam	Anti-m^6A antibody produced in mouse	ab151230	RIP: 5μg
Cell Signaling Technology	Anti-AGO2 antibody produced in rabbit	2897	RIP: 5μg
